# Local Spinal Cord Injury Treatment Using a Dental Pulp Stem Cell Encapsulated H_2_S Releasing Multifunctional Injectable Hydrogel

**DOI:** 10.1002/adhm.202302286

**Published:** 2023-12-16

**Authors:** Abdullkhaleg Ali Albashari, Yan He, Yu Luo, Xingxiang Duan, Jihea Ali, Mingchang Li, Dehao Fu, Yangfan Xiang, Youjian Peng, Song Li, Lihua Luo, Xingjie Zan, Tushar Kumeria, Qingsong Ye

**Affiliations:** ^1^ Center of Regenerative Medicine, Department of Stomatology Renmin Hospital of Wuhan University Wuhan Hubei 430060 China; ^2^ School and Hospital of Stomatology Wenzhou Medical University Wenzhou Zhejiang 325035 China; ^3^ Laboratory for Regenerative Medicine Tianyou Hospital Wuhan University of Science and Technology Wuhan Hubei 430064 China; ^4^ Oral Maxillofacial Department Massachusetts General Hospital Harvard Medical School Boston MA 02114 USA; ^5^ College of Life and Environmental Science Wenzhou University Wenzhou Zhejiang 325035 China; ^6^ Department of Neurosurgery Renmin Hospital of Wuhan University Wuhan Hubei 430060 China; ^7^ Department of Orthopaedics Shanghai Sixth People's Hospital Shanghai Jiao Tong University Shanghai 200233 China; ^8^ Wenzhou Institute University of China Academy of Science Wenzhou Zhejiang 325024 China; ^9^ School of Materials Science and Engineering University of New South Wales Sydney New South Wales 2052 Australia; ^10^ Australian Center for NanoMedicine University of New South Wales Sydney New South Wales 2052 Australia; ^11^ School of Pharmacy University of Queensland Brisbane Queensland 4102 Australia

**Keywords:** controlled drug delivery, mesoporous silica nanoparticles, spinal cord injuries, stem cell therapy, thermoresponsive hydrogel

## Abstract

Spinal cord injury (SCI) commonly induces nerve damage and nerve cell degeneration. In this work, a novel dental pulp stem cells (DPSCs) encapsulated thermoresponsive injectable hydrogel with sustained hydrogen sulfide (H_2_S) delivery is demonstrated for SCI repair. For controlled and sustained H_2_S gas therapy, a clinically tested H_2_S donor (JK) loaded octysilane functionalized mesoporous silica nanoparticles (OMSNs) are incorporated into the thermosensitive hydrogel made from Pluronic F127 (PF‐127). The JK‐loaded functionalized MSNs (OMSF@JK) promote preferential M2‐like polarization of macrophages and neuronal differentiation of DPSCs in vitro. OMSF@JK incorporated PF‐127 injectable hydrogel (PF‐OMSF@JK) has a soft consistency similar to that of the human spinal cord and thus, shows a high cytocompatibility with DPSCs. The cross‐sectional micromorphology of the hydrogel shows a continuous porous structure. Last, the PF‐OMSF@JK composite hydrogel considerably improves the in vivo SCI regeneration in Sprague–Dawley rats through a reduction in inflammation and neuronal differentiation of the incorporated stem cells as confirmed using western blotting and immunohistochemistry. The highly encouraging in vivo results prove that this novel design on hydrogel is a promising therapy for SCI regeneration with the potential for clinical translation.

## Graphical abstract

A thermoresponsive hydrogel is designed for a dual‐pronged local therapy of spinal cord injuries (SCI). The composite hydrogel system can deliver H_2_S gas as well as encapsulating stem cells and enables improved SCI healing through precise modulation of injury site inflammation as demonstrated by a thorough in vitro and in vivo investigation.

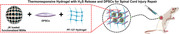

## 1 Introduction

Spinal cord injuries (SCI) are appraised as one of the most serious neuronal degradation challenges worldwide.^[^ ^1^ ^]^ SCI leads to axonal degeneration and neuronal death, which cause temporary or permanent nerve dysfunction and loss of sensation at the injury site.^[^ ^2^ ^]^ After the injury, several complex biochemical processes take place at the site of injury such as apoptosis, oxidative stress, and inflammation. The inflammatory response during the initial stage of the injury is the main factor that inhibits neural differentiation of stem cells and affects the nerve repaired by promoting cell apoptosis, and degeneration of the nerve. Besides, the lack of neural cell differentiation in the SCI can also cause nerve regeneration failure.^[^ ^3^ ^]^ Subsequently, inhibition of the inflammatory factors and guaranteeing neural cells growth factors can improve the regenerative microenvironments, ultimately leading to restored nerve function and activity.^[^ ^4^ ^]^ The advances in cell therapy technology have shown strong potential for efficient SCI treatment.^[^ ^5^ ^]^ So far, the most widely used strategy is centered around enhancement in neural stem cell differentiation by combining scaffolds with growth factors or drugs. This strategy also supports tissue microenvironment and repairs damaged SCI sites.^[^ ^6^ ^]^ For example, our group designed a novel thermosensitive hydrogel containing dental pulp stem cells (DPSCs) with basic fibroblast growth factor (bFGF), which can be easily delivered into the SCI to ensure the high density of DPSCs and the sustained release of bFGF during recovery process.^[^ ^7^ ^]^

Hydrogen sulfide (H_2_S) has been proven as a unique endogenous gaseous transmitter.^[^ ^8^ ^]^ Many experiments confirmed H_2_S simulated tissue and biological functions in the human body, such as inhibiting the inflammatory response,^[^ ^9^ ^]^ and importantly, expanding plasma IL‐10 levels, while suppressing the accumulation of lipopolysaccharide (LPS)‐induced neutrophils plasma interleukin‐1 (IL‐1β),^[^ ^10^ ^]^ and tumor necrosis factor‐α (TNF‐α).^[^ ^11^ ^]^ Hydrogen sulfide is not applied in the clinic directly because of the high toxicity associated with the uncontrollable release of this gas. In this regard, H_2_S donors have emerged as potential convenient substitutes for direct H_2_S dosing.^[^ ^12^ ^]^ Sodium Hydrosulfide (NaSH) is one of the commonly used donors of H_2_S. However, NaSH releases H_2_S too fast to be used for biomedical applications. Lawesson's reagent is another H_2_S donor, which spontaneously released H_2_S in an aqueous solution. However, its limited aqueous solubility hinders its use in medicine.^[^ ^13^ ^]^ Based on Lawesson's reagent, Moore prepared a novel water‐soluble donor called GYY4137 in 2008,^[^ ^13^ ^]^ and studies displayed GYY4137 donor has multivalent anti‐thrombosis, anti‐tumor, anti‐shock, and anti‐inflammation functions.^[^ ^14^ ^]^ The GYY4137 approach displayed neuroprotective outcomes in SCI of diabetic models and altered their sensory deficiencies.^[^ ^15^ ^]^ Both GYY4137 and NaHS were found to inhibit LPS‐induced NF‐κB receptor activation and release of IL‐1β and tumor necrosis factor alpha (TNF‐α) from the macrophage. Furthermore, H_2_S attenuated with LPS and studying p38 MAPK phosphorylation in BV‐2 cells (Microglial cells) resulting in anti‐inflammatory products such as inducible nitric oxide synthase, nitric oxide (NO), and TNF‐α.^[^ ^16^ ^]^ After these findings, a series of novel H_2_S donors have been prepared based on the phosphonamidothioate such as JK1, JK2, and JK3.^[^ ^17^ ^]^ Related to other known H_2_S donors, these have considerable advantages including high aqueous solubility, slow and controlled production of H_2_S, and pH‐responsive H_2_S release. However, these JK class of H_2_S donors tend to evolve H_2_S faster at lower pH, which makes them unsuitable for localized application at the SCI site with sustained inflammation.^[^ ^18^ ^]^ Therefore, advanced nano‐drug delivery systems that can enable controlled release of H_2_S from such donors at the inflamed SCI site hold tremendous clinical translational potential.

Recently, nanomaterials such as metal/metal oxide.^[^ ^19^ ^]^ polymer,^[^ ^20^ ^]^ mesoporous silica (MSNs).^[^ ^21^ ^]^ and carbon nanotubes.^[^ ^22^ ^]^ have been employed as drug carriers.^[^ ^23^ ^]^ Among these, MSNs have emerged as one of the ideal drug carriers, because of unique properties such as highly tunable particle size, biocompatibility, high specific surface area, easy and widely available surface chemistry, and large pore volume. These features translate to ultra‐high drug loading capacity, protection of drug payload, and controlled release due to confinement of drug in the mesopores of MSNs confined,^[^ ^24^ ^]^ which may safely deliver nanoparticles therapeutic factors to specific cells and injury sites.^[^ ^25^ ^]^ In addition, clever modification of MSNs has led to the generation of stimuli‐responsive MSN nano‐carriers, with a range of available stimuli enzyme, heat, magnetic field, pH‐sensitive, redox reaction, magnetic field, etc.^[^ ^26^ ^]^ However, most of these systems are designed for intravenous delivery of drugs through a stimulus from either an endogenous or an exogenous source. Thus, making these unsuitable for application in SCI where a sustained and controlled dose of therapeutic payload is required locally.

**Scheme 1:**
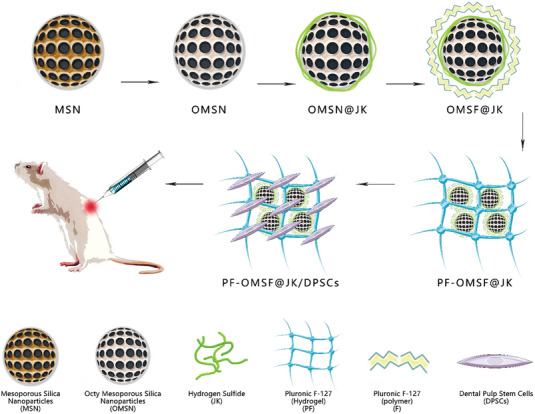
A schematic showing a step‐by‐step process of generating a multifunctional thermoresponsive hydrogel with stem cell delivery and H_2_S releasing capabilities. Octyl functionalized MSN (OMSN) particles modified with a Pluronic F‐127 (PF‐127) overcoat were used as a carrier to incorporate JK, an H_2_S gas donor molecule, into the PF‐127 polymer‐based thermoresponsive hydrogel. The hydrogel allowed the incorporation of DPSCs for local delivery of both H_2_S gas and stem cells to the SCI site through injection.

In this work, a novel composite injectable hydrogel is prepared for multidimensional localized SCI therapy through the delivery of stem cells and sustained release of H_2_S gas. The sustained release of H_2_S is enabled by the JK (an H_2_S donor) loaded MSNs, which serve as nano‐depots of the therapeutic gas from the hydrogel. As illustrated in **Scheme** **1**, JK‐loaded Octyltriethoxysilane functionalized MSN (OMSN@JK) were modified with PF‐127 (OMSF@JK) to enable controlled release of the H_2_S gas and easy incorporation of hydrophobic OMSF@JK into the aqueous hydrogel. The OMSF@JK particles were incorporated into a stimuli‐responsive Pluronic F‐127 (PF‐127)‐based hydrogel at 17 wt% loading, forming PF‐OMSF@JK with localized H_2_S release and thermoresponsive gelling features. Highly biocompatible components of PF‐OMFS@JK hydrogel allowed encapsulation and a high survival rate of dental pulp‐derived stem cells (DPSCs). The presented PF‐OMSF@JK/DPSCs multifunctional hydrogel offers several advantageous properties that make it ideal for localized therapy of SCI: 1) preservation of the activity of JK and DPSCs, 2) high hydrogel conductivity, 3) improved mechanical properties of hydrogel through favorable interaction between the OMSF@JK particles and the PF‐127‐based hydrogel, and 4) easy application at the SCI site due to liquid‐to‐gel switching properties of the PF‐OMSF@JK/DPSCs.

## 2 Results

### 2.1 Characterization of Nanoparticles

**Figure 1:**
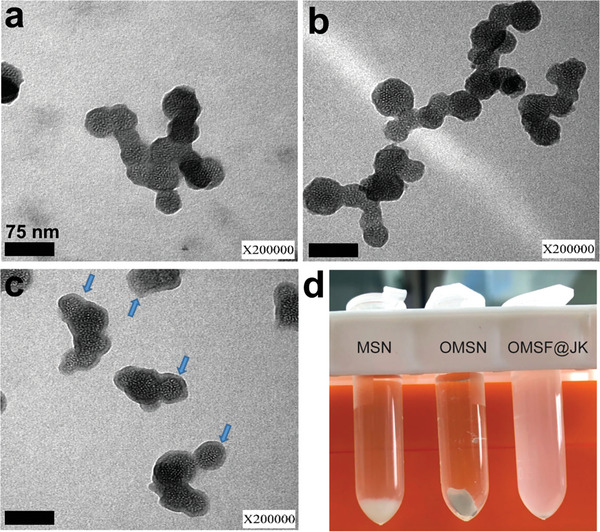
TEM images showing the morphology of a) MSN, b) OMSN, and c) OMSF@JK. MSN and OMSN showed an aspherical shape (30–50 nm in size) with a visible porous structure and maintained the surfaces‐porous in OMSN: and coated overall shape in OMSF@JK with a thin layer containing JK and PF‐127 (Scale bar: 75 nm). d) Digital photographs of particles suspended in PBS (pH 7.4). MSN, OMSN, and OMSF@JK were suspended in PBS at 1 mg mL^−1^ concentration ultrasonically (5 min, 80 w, water bath) and photographs were taken after 30 min of incubation at room temperature.

TEM images of MSN and OMSN in **Figure** **1**a,b, respectively, show no major morphological change in MSN after OTS functionalization with particle size ranging between 30 and 50 nm size for MSN. While the OMSN (Figure 1b) maintains the particle size there is an observed increase in porosity (Figure S1a, Supporting Information). The OMSF@JK nanoparticles appear softly mottled on the outer surface in contrast to the OMSN. The OMSF@JK has a thin layer of PF‐127 around OMSN, which was expected. The thickness of the PF‐127 layer was measured to be around 3–6 nm (Figure 1c). TGA analysis presented in Figure S1b, Supporting Information shows a weight loss for the MSN, OMSN, and OMSF@JK with 83.072%, 83.494%, and 66.691%, of the initial mass preserved upon heating to 600 °C at an N_2_ atmosphere. As expected, the higher weight loss after each modification further confirmed the successful surface modification with OTS and the loading of JK into OMSN. The TGA data also confirmed that OMSN achieved ≈16.8 wt% loading of JK. In Fourier transform infrared (FTIR) spectra presented in Figure S1c, Supporting Information, MSN exhibits a characteristic peak of CH medium stretch (3742 cm^−1^), CH_2_ weak stretch (2935 cm^−1^), C═C strong stretch scissor (2366 cm^−1^), C═O weak stretch (1970 cm^−1^), C─C medium stretch (1559 cm^−1^), C─H strong stretch (1086 cm^−1^), C─C weak anti symmetric bending (964 cm^−1^), and C─H medium stretch (799 cm^−1^). Also, OMSN exhibits a characteristic peak of CH weak stretch (3751 cm^−1^), CH_3_ weak stretch (2961 cm^−1^), C═C medium stretch scissor (2366 cm^−1^), C═C medium stretch scissor (2157 cm^−1^), C═O weak stretch (1636 cm^−1^), C─H strong stretch (1086 cm^−1^), and C─H medium stretch (799 cm^−1^). OMSF@JK exhibits a characteristic peak of CH weak stretch (3744 cm^−1^), CH_3_ medium stretch (2961 cm^−1^), C═C medium stretch scissor (2366 cm^−1^), C═C medium stretch anti symmetric bending (2161 cm^−1^), C═O weak stretch (1636 cm^−1^), C─O medium stretch (1454 cm^−1^), C─H strong stretch (1086 cm^−1^), and C─H medium stretch (798 cm^−1^). Furthermore, successful OMSF@JK modification was proved by the disappearance of the signal at 2880 cm^−1^ in the FTIR data. A digital photograph of the three samples is provided in Figure 1d, OMSF@JK showed good dispersibility in PBS (pH = 7.4) at a 1 mg mL^−1^ concentration with minor aggregation. However, MSN and OMSN precipitated and aggregated in PBS at the same concentration after 30 min of incubation at room temperature.

### 2.2 Morphological and Phenotypic Characteristics of Harvested DPSCs

To ensure stem cell phenotypic characteristics were maintained by the extracted DPSCs, their morphology, and MSCs‐like surface markers were analyzed. The morphological evolution of the DPSCs through different passages over 14 days is presented in Figure S2a, Supporting Information. The shrinkage of the cell cluster and evolution from a cluster into fibroblast‐like cells is evident from days 0 to 7 (second passage), while at day 14 (third passage) the cell cluster disappears completely with well‐attached and spread‐out cells with fibroblast‐like morphology covering the whole flask. In the third passage, to examine the MSCs‐like identification and characteristics of DPSCs the multilineage flow cytometry was implemented. The flow cytometric results indicated that DPSCs negatively expressed the surface markers associated with aged hematopoietic pulp stem cells such as CD19, HLA‐DR, and CD14, while positively expressing MSC‐like phenotypic markers CD90 (Figure S2b, Supporting Information).

### 2.3 Cytocompatibility of Nanoparticles

First, the effect of different concentrations of OMSN and OMSF on cell viability was evaluated. The result showed that even at high concentrations (up to 600 µg mL^−1^), OMSN and OMSF were well‐tolerated by DPSCs (Figure S3a, Supporting Information). Also, the effects of various concentrations of pure JK and OMSF@JK (0, 3, 5, 15, 30, 40, 60, and 100 µg mL^−1^) on the cell viability were assessed over a period of 3 days. Results from the CCK‐8 assay showed that lower doses of OMSF@JK improved the cell viability of DPSCs on days 1 and 3 (Figure S3b,c, Supporting Information). Lower concentrations of up to 60 µg mL^−1^ of OMSF@JK yielded conducive and highly compatible conditions for DPSCs in comparison with the same concentration of pure JK as shown in Figure S3b,c, Supporting Information.

### 2.4 Impact of OMSF@JK on the Neurogenic Differentiation of DPSCs

The neuronal differentiation efficiency of OMSF@JK on DPSCs under LPS activation was assessed at different doses ranging between 30 and 100 µg mL^−1^. DPSCs treated with all the OMSF@JK concentrations appear long‐spindle‐like by day 3 with differentiation continuing over day 6 that peaked by day 12 when the majority of DPSCs assumed multiple cellular appearances extending the cell bodies (Figure S4, Supporting Information). Also, small flat, round oligodendrocyte‐like cells were seen in OMSF@JK treated DPSCs groups. Whereas, some fibroblast‐like DPSCs with spread‐out shapes were observed in neurogenic media (NM) and NM/LPS. The results show that the DPSCs at day 12 displayed a classical neuronal cell morphology with long and thin cytoplasmic processes, dendrite and axon presence, and perikaryon upon treatment with OMSF@JK.

To characterize the stemness of DPSC, the cells were stained with the fluorescently labeled CD 146 marker antibody. The fluorescence (FL) intensity of CD 146 was quantified and compared with the control group. The CD 146 expression was considerably lower in all the groups, relative to the marker for MSCs (Figure S5a, Supporting Information). The FL intensity of anti‐GFAP was quantified and compared with the LPS group with the data in Figure S5b, Supporting Information showing significantly higher anti‐GFAP FL intensity for OMSF@JK1 and OMSF@JK2 treated cells. To characterize neural differentiation, the cells were stained with markers against MAP‐2 and Anti‐GFAP over 12 days. Immunofluorescence analyses indicated strong expressions of MAP‐2 on day 6 (Figure S5c, Supporting Information), which suggests that cells changed bipolar morphology for all OMSF@JK treated groups. Normalized FL intensity of MAP‐2 was quantified and compared against that of the NM‐LPS group (Figure S5d, Supporting Information). The MAP‐2 and anti‐GFAP expressions were significantly higher in OMSF@JK1, OMSF@JK2, and OMSF@JK3 (Figure S5d, Supporting Information). At day 12, the MAP‐2 expression was suggestively greater in OMSF@JK1, OMSF@JK2, and NM‐LPS than in the OMSF@JK3 group (Figure S5e, Supporting Information). The fluorescence intensity of anti‐GFAP was quantified and compared with the NM group, which also showed significantly higher fluorescence from OMSF@JK1, OMSF@JK2, and OMSF@JK2 treated cells (Figure S5f, Supporting Information). Finally, the data exposed advanced enhancement in MAP‐2 and Anti‐GFAP in OMSF@JK groups.

The qRT‐PCR analyses of neural marker expression by DPSCs were also carried out on day 12. As shown in Figure S6, Supporting Information, DPSCs treated with LPS and different concentrations of OMSF@JK displayed significantly upregulated expression of Nestin, NeuroD1, Fibronectin, and MAP‐2. Furthermore, significant changes in the expression of Nestin were observed in all treatment groups compared with the NM and control group after day 12 of neural induction (Figure S6a, Supporting Information). Also, NeuroD1 expression (Figure S6b, Supporting Information) was observed to be significantly upregulated in OMSF@JK2 treated compared to the OMSF@JK1, LPS, and NM treatment group. OMSF@JK2 treated exhibited significantly increased expression of Fibronectin, as compared to the control, whereas OMSF@JK1 and OMSF@JK3 treated displayed significantly decreased expression compared to OMSF@JK2 (Figure S6c, Supporting Information). However, MAP‐2 was observed to be significantly upregulated in NM/LPS and OMSF@JK co‐treatment groups, but not in NM and control (Figure S6d, Supporting Information).

### 2.5 Anti‐Inflammatory Properties of Nanoparticles

To understand the inflammatory effects of the OMSF@JK, the macrophage cells (RAW 264.7) were co‐treated with LPS and different concentrations of OMSF@JK for 24 h. The cells were analyzed for their morphological changes by SEM and quantification of cell surface markers through immunofluorescence and gene marker expression measurements via PCR of key factors associated with inflammation. Macrophages cultured in an LPS‐containing medium showed clustered and round morphology. With an increase in OMSF@JK concentration, a more elongated shape, and loosely compact macrophage morphology was observed compared to the LPS group (Figure S7a,b, Supporting Information), which hallmarks M2‐like macrophages with anti‐inflammatory properties. Quantification of immunofluorescence signal in the confocal microscopy images presented in Figure S7b, Supporting Information revealed a significant downregulation of IL‐6 expression in OMSF@JK‐treated treated cells compared to the LPS‐treated group (Figure S7c, Supporting Information). Gene expression measurements for iNOS (Figure S7d, Supporting Information), TNF‐α (Figure S7e, Supporting Information), ARG1 (Figure S7f, Supporting Information), and CD163 (Figure S7g, Supporting Information) macrophage phenotype markers were determined by PCR. The gene expression of iNOS and TNF‐α, associated with M1‐like macrophages, was downregulated in the OMSF@JK‐treated groups compared to the LPS groups (Figure S7d,e, Supporting Information, respectively). By contrast, the M2‐like macrophage marker genes ARG1 were upregulated in all the OMSF@JK treated group (Figure S7f,g, Supporting Information, respectively). The expression of the CD163 gene, another M2‐like macrophage marker was high for OMSF@JK treated cells compared to the LPS‐treated cells. Interestingly, only OMSF@JK2 showed a higher CD163 expression indicating the key role of H_2_S dose in regulating the immune behavior of macrophages. As a confirmatory proof of the anti‐inflammatory properties of OMSF@JK, the expression of IL‐10 which is a well‐known marker of M2‐like macrophage phenotype was assessed using immunofluorescence labeling (Figure S8, Supporting Information). The results show an increase in IL‐10 expression with increasing OMSF@JK treatment concentration.

### 2.6 Hydrogel Characterization

**Figure 2:**
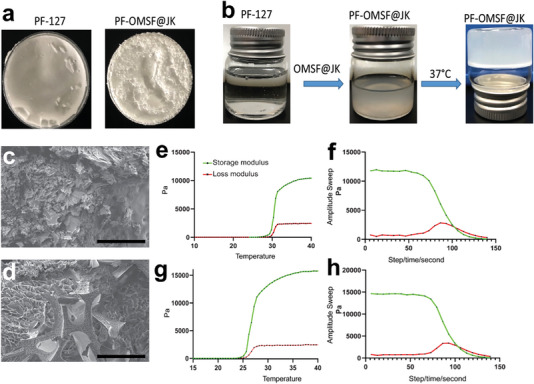
Characterization of hydrogels: a) Image of the dried hydrogels (Scale bar: 1 cm). b) Image of the hydrogels (Scale bar: 1 cm). c) An SEM image showing the micromorphology of the PF‐127 with porous structural features. The interconnection pores in the PF‐127 hydrogel appeared flattened in shape. d) An SEM image of dried PF‐OMSF@JK with similar porous microstructural features. At low magnification, representative SEM images of PF‐OMSF@JK hydrogel showed a deep and irregular rounder shape. e) The gelation temperature of PF‐127 hydrogel was determined by rheological measurements showing a sol‐to‐gel transition around 28 °C. f) The storage moduli and loss moduli of angular frequency for PF127 at 37 °C. g) The gelation temperature of PF‐OMSF@JK hydrogel was reduced to around 24 °C as determined by rheological measurements. h) The storage moduli and loss moduli of angular frequency for PF‐OMSF@JK at 37 °C. It represents that the storage modules of PF‐OMSF/JK are higher than the loss with a linear character in a small range of strain amplitudes. Sand PF‐OMSF@JK hydrogels: at high magnification, the PF‐OMSF@JK hydrogel showed a smaller porous structure and a slightly denser compared with the PF‐127.

Digital photographs of the dried form of blank PF‐127 hydrogel and OMSF@JK incorporated PF‐127 (PF‐OMSF@JK) hydrogel are provided in **Figure** **2**a. Whereas, Figure 2b shows the hydrated form of the PF‐127 gel (left image) and PF‐OMSF@JK (middle image) at room temperature. The right image in Figure 2b shows the solidification of the PF‐OMSF@JK upon heating to 37 °C. SEM images of dried PF‐127 and PF‐OMSF@JK are provided in Figure 2c,d, respectively, that prove no structural changes take place upon the addition of OMSF@JK into the PF‐127 hydrogel. Although both the dried hydrogels show porous features, such features might not exist in the hydrated form of the hydrogel. PF‐OMSF@JK hydrogel presented a lightly denser and smaller porous structure.

It is important to study the rheomechanical properties of the thermosensitive hydrogel prior to application at SCI site. The temperature‐dependent rheological measurement of storage (G′) and loss modulus (G′′) (Figure 2e,f) were used to determine the gelation temperature. The G′ intersection of G′′ curves outlined the transformation at gelation temperature, which was found to be around 28 °C for the blank PF‐127 (left image, Figure 2e) hydrogel and around 24 °C for PF‐OMSF@JK (right image, Figure 2f). In addition, compared to the blank PF‐127 hydrogel (left image, Figure 2g) the storage modulus of PF‐OMSF@JK (right image, Figure 2h) was considerably higher. This could be attributed to stronger hydrophobic interactions between the eight‐carbon long hydrocarbon chain of the octyltriethoxysilane on MSN and the hydrophobic part of the PF‐127 triblock polymer and the Schiff base linkages.

### 2.7 In Vitro H_2_S Release

This novel composite of PF‐127 hydrogel with specifically functionalized MSN particles to incorporate JK, an H_2_S donor, allows for a pH‐dependent H_2_S release at the site of gel applications. Following evaluation of the H_2_S release from PF‐OMSF@JK and control at two different pH values (pH 6.4 and 7.4). As presented in (Figure S9a, Supporting Information), both PF‐JK and PF‐OMSF@JK hydrogel showed a faster release of H_2_S at pH 6.4 with a considerable burst release of H_2_S (almost 80 µm) over the first few hours that reduced to around 20 µm over the 60 h duration of the total release measurements. Particularly, the peak time in the PF‐JK group for pH 6.4 was around 60 min, while for PF‐OMSF@JK was delayed to around 180 min (Figure S9a, Supporting Information) with a further sustained release for up to 6 h which is a significant improvement compared to when JK is directly incorporated into the hydrogel. The H_2_S release half‐life was prolonged almost 1.2 folds, contrasted with JK alone in the PF‐127 hydrogel. At pH 7.4 (Figure S9b, Supporting Information), H_2_S burst in the first few hours of incubation was not observed. Instead, an almost zero‐order release was evident over the first 24 h followed by plateauing of the H_2_S release. At this pH, PF‐OMSF@JK released an overall lower amount of H_2_S relative to the control (Figure S9b, Supporting Information). This data indicated that PF‐OMSF@JK could appreciably extend the H_2_S evolution time for more suitable and prolonged localized H_2_S treatment, which was also dependent on the local pH.

### 2.8 DPSCs Encapsulation and Compatibility with Hydrogels

To ensure the viability of DPSCs embedded into the hydrogels, in vitro DPSCs viability and proliferation behavior in response to PF‐127 and PF‐OMSF@JK2 was evaluated. The hydrogel did not modify the DPSCs viability up to 3 days in culture (day 1 shown in Figure S10a, Supporting Information, and day 3 shown in Figure S10b, Supporting Information) as measured by CCK‐8 assay. As shown in (Figure S10a,b, Supporting Information), DPSCs demonstrated high viability on PF‐OMSF@JK2 for the whole period, relative to tissue‐culture plastic control. The proliferation of the DPSCs was measured through laser scanning confocal microscopy (LSCM) both PF‐127 and PF‐OMSF@JK2 promoted strong DPSCs proliferation as seen in the 3D render shown in Figure S10c, Supporting Information. The cell nucleus was labeled with DAPI, while the cytoskeleton was fluorescently stained with TRITC‐tagged phalloidin. The cells appear to be well dispersed within the 3D hydrogel volume.

### 2.9 In Vivo Inflammation Reduction at SCI Site

**Figure 3:**
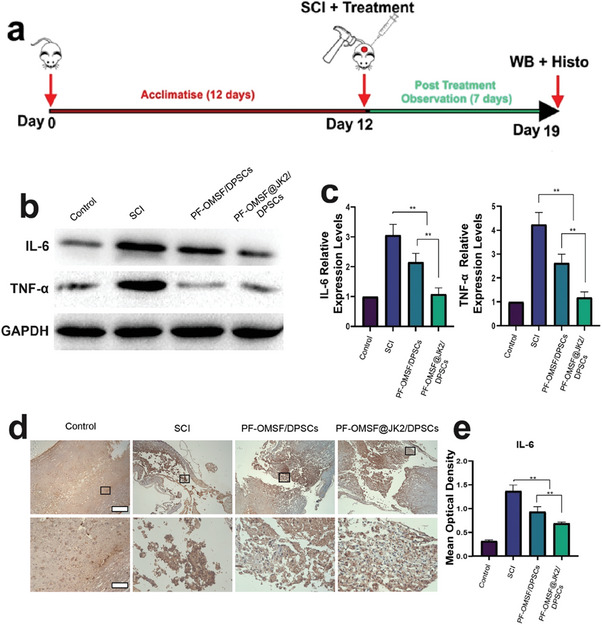
Reduction of pro‐inflammatory cytokines in hydrogel‐treated SCI rats: a) Experimental timeline showing period of acclimatization of 12 days followed by creation of SCI and treatment. The rats were observed and sacrificed for terminal assays 7 days post treatment. b) WB of the protein expression of IL‐6 and TNF‐α on the 7th day after spinal cord injury. c) Quantification of the WB of IL‐6 and TNF‐α on the 7th day. These showed that PF‐OMSF@JK treatment was the most effective intervention, where the expression of the pro‐inflammatory factors, IL‐6 and TNF‐α, was significantly decreased compared to the injury group and there was a similar expression level between control and PF‐OMSF@JK treatment. d) Cross‐sectioning results of the protein expression of IL‐6 via the immunohistochemical staining on the 7th day of SCI. e) Quantification of the immunohistochemical staining of IL‐6, where the expression of IL‐6 was significantly downregulated in PF‐OMSF@JK/DPSCs compared to the SCI and was similar expression IL‐6 level between control and PF‐OMSF@JK treatment. Scale bar: 500 µm (upper panel) and 100 µm (lower panel) *p < 0.05, **p < 0.01.

To evaluate the pro‐inflammatory cytokine reduction capabilities of OMSF@JK2/DPSCs, expression levels of IL‐6 and TNF‐α that are two key inflammation driving cytokines were measured in rats at day 7 after the induction of SCI by Western Blotting and the protein expression quantification. At day 7, the gene expression of IL‐6 and TNF‐α and levels were increased in the SCI group in comparison with the control group (*P* < 0.05, **Figure** **3**a,b). It is worth noting that PF‐OMSF@JK2 hydrogel embedded with DPSCs (PF‐OMSF@JK2/DPSCs) displayed the most pronounced IL‐6 and TNF‐α decrease, relative to the untreated SCI animal group (Figure 3a,b). Excitingly, the levels of IL‐6 and TNF‐α for the PF‐OMSF@JK2/DPSCs were comparable to the healthy animal groups.

Immunohistochemical staining showed that the PF‐OMSF@JK2/DPSCs treated animals exhibited an important decrease in IL‐6 staining density at the SCI site compared to the untreated SCI control (Figure 3c). This purported that PF‐OMSF@JK2/DPSCs efficiently decreased the SCI site inflammation. Quantification of the optical density in the immunohistochemistry staining (Figure 3d) revealed the same outcome with a definite difference between PF‐OMSF@JK2/DPSCs treated SCI animals and untreated SCI animals. Like the protein expression levels determined using Western blotting, the optical density of the cells positive to IL‐6 was almost half in animals treated with PF‐OMSF@JK2/DPSCs hydrogel in comparison to the untreated SCI animals. The IL‐6‐stained cell number for PF‐OMSF@JK2/DPSCs was comparable to the healthy animals (Figure 3d). In addition, the hematoxylin and eosin (H&E) stained histological sections of the whole spinal cord from different treatment groups are included in Figure S11, Supporting Information along with their corresponding high‐magnification images. The proximal, epi‐center, and distal ends of the spinal cord section are visible in the full‐scale spinal cord sections. The H&E stained whole spinal cord sections provide a comprehensive view of the entire tissue showing a large lesion cavity for the untreated animal (SCI group). The PF‐OMSF//DPSCs and PF‐OMSF@JK2/DPSCs groups showed a highly reduced lesion cavity (Figure S11, Supporting Information).

### 2.10 Microglia/Macrophages Activation Properties of the Hydrogels

**Figure 4:**
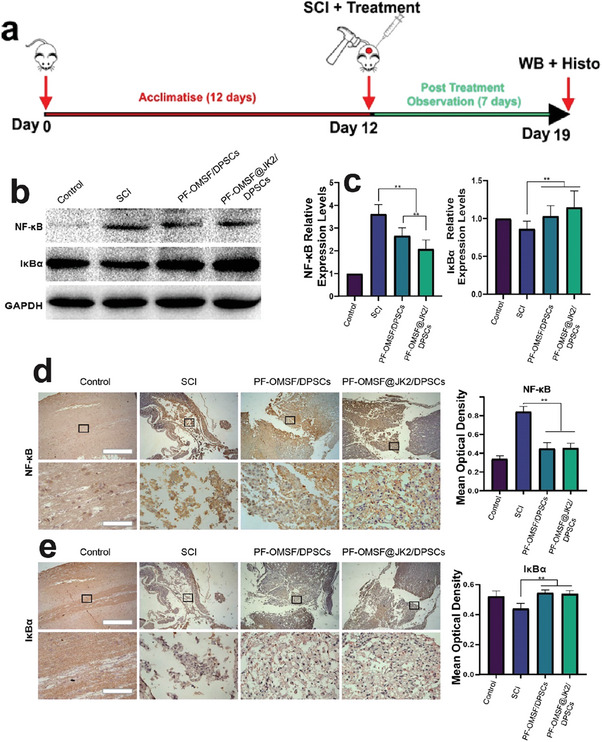
Prevented microglia/macrophages activation properties of the hydrogels: a) WB of the protein expression of NF‐κB and IκB‐α on the 7th day after spinal cord injury. b) Quantification of the WB of NF‐κB and IκB‐α on the 7th day. c) Cross‐sectioning results of the protein expression of NF‐κB via the immunohistochemical staining on the 7th day of SCI and quantification of the immunohistochemical staining of NF‐κB. d) Cross‐sectioning results of the protein expression of NF‐κB via the immunohistochemical staining and quantification analysis Scale bar: 500 µm (upper panel) and 100 µm (lower panel). e) Cross‐sectioning results of the protein expression of IκB‐α via the immunohistochemical staining and quantification analysis. Scale bar: 500 µm (upper panel) and 100 µm (lower panel). *p < 0.05, **p < 0.01.

Alongside the reduction of pro‐inflammatory cytokines, further assessments were done to measure the effect of PF‐OMSF@JK2/DPSCs on anti‐inflammatory cytokines at the SCI site. This was carried out by measuring the NF‐κB levels by Western blot and immunohistochemistry analysis at the SCI site 7 days post treatment (study design in **Figure** **4**a). As shown in Figure 4b, on day 7 the NF‐κB expression levels were notable at the SCI site for all three sample groups (i.e., untreated SCI, and PF‐OMSF/DPSCs and PF‐OMSF@JK2/DPSCs treated SCI) compared to the healthy animals showing inflammation and an acute state of the injury. In PF‐OMSF@JK2/DPSCs, the protein expression of NF‐κB was significantly decreased compared to the untreated SCI and PF‐OMSF/DPSCs group (Figure 4c, left bar‐graph). The PF‐OMSF@JK2/DPSCs promoted an early NF‐κB inhibition. Also, the PF‐OMSF@JK2/DPSCs group showed a noticeable increase in protein expressions of IκB‐α correlated to the SCI (Figure 4c right bar‐graph), relative to the untreated SCI group. However, the IκB‐α levels for both the hydrogels (i.e., with or without JK) were comparable. Immunohistochemistry staining and analyses of NF‐κB and IκB‐α (Figure 4d,e) expressions on day 7 of the treatment were carried out similarly to the IL‐6 and TNF‐α. The NF‐κB was highly expressed in the untreated SCI animal group, pointing to an inhibited regeneration at the injury site. The PF‐OMSF@JK2/DPSCs group presented an increased IκB‐α expression stain and a lower NF‐κB expression which are typical of a tissue‐repair‐friendly microenvironment (Figure 4d,e).

### 2.11 Neurite and Improved Cell Sprouting with Cell Encapsulated Hydrogel

**Figure 5:**
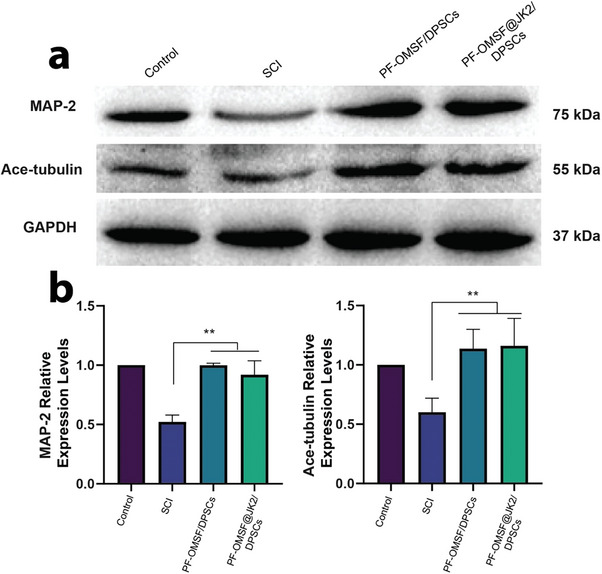
Promoted neurite and improved cell sprouting properties of the hydrogels: a) WB of the protein expression of microtubule‐associated protein 2 (MAP‐2) (75 kDa) and acetylated tubulin (Ace‐tubulin) 55 kDa on day 7 post‐SCI. b) Quantification of the WB of MAP‐2and Ace‐tubulin on the 7th day. *p < 0.05, **p < 0.01.

The expression of microtubule associated protein 2 (MAP‐2) and acetylated tubulin (Ace‐tubulin) on day 7 post‐SCI in the dissected injury tissue was examined. Ace‐tubulin may perform a significant part during microtubule structure‐function and neural differentiation; connected with microtubule‐associated protein, MAP‐2 is an essential factor to stabilize the nerve microtubules. Western blot data presented a decreased Ace‐tubulin and MAP‐2 protein expression in the untreated SCI group (**Figure** **5**a). In contrast, all treatment groups displayed remarkably high protein expression of MAP‐2 and Ace‐tubulin (*P* < 0.01), with the PF‐OMSF@JK2/DPSCs showing the highest expression. Thus, PF‐OMSF@JK2/DPSCs represented an effective increase in protein expression of neural repair (Figure 5a,b).

## 3 Discussion

SCI can cause serious sensory‐motor, and dysautonomia.^[^ ^1b^ ^]^ The delivery of drugs drug at the SCI site is still a major challenge in SCI therapy because of the degradation and rapid metabolism in blood and plasma clearance in the injury site, and poor diffusion into the intercellular membrane.^[^ ^27^ ^]^ It has been shown that nanomedicine therapy might encourage spinal cord treatment and good efficacy of drug due to improving drug pharmacokinetic propitiates and better cell membrane integration.^[^ ^28^ ^]^ However, different nanocarriers demand a combination of functional design features to obtain targeted drug delivery, which can prevent pharmaceutical drug development and their clinical translation.^[^ ^29^ ^]^ In this study, we developed OMSF as the drug carrier. The size of the OMSF@JK nanoparticles was controlled to be around 30–50 nm (Figure 1). We prepared the OMSF@JK composite nanoplatforms for SCI. Previous data showed that the recommended nanoparticle localization to SCI is below 200 nm.^[^ ^30^ ^]^ The nanoparticles we prepared to meet those requirements (Figure 1c,d). Functionalization of MSN with Octylsilane was strategically carried out to enable a strong cross‐link between the OMSF particles and PF‐127.^[^ ^26^, ^31^ ^]^

The results of the CCK‐8 assay indicated that JK and OMSF@JK have no obvious toxic influence on the proliferation of DPSCs cells. Previous data showed that H_2_S donors played a great neuroprotective factor and the basic mechanisms were due to its anti‐apoptotic and anti‐inflammatory effects.^[^ ^32^ ^]^ Lately, it has been discovered that H_2_S donors can act as an inducer for neuronal differentiation of NG108‐15 cells.^[^ ^33^ ^]^ Besides, H_2_S at physiological concentrations could be increased during hepatic differentiation in DPSCs. More importantly, the moderating effect of H_2_S on the differentiation of progenitor cells in other tissues has also been reported.^[^ ^34^ ^]^ In this study, JK, an H_2_S donor with sufficient aqueous solubility was loaded onto OSMF particles. Our data suggested that OMSF@JK could improve DPSCs proliferation in vitro. Also, OMSF@JK encouraged neuronal differentiation of DPSCs, which was related to the altered expression of neuronal differentiation genes. Moreover, H_2_S stimulated released from the OMSF@JK even promoted neurogenesis properties in the DPSCs with LPS activation.

RAW264.7 cells, as a prominent macrophage cell type in acute SCI, are known to play an important role in tissue regeneration. Different macrophage cell phenotypes have been studied deeply in relation to neuroregeneration and remyelination.^[^ ^35^ ^]^ The RAW cells have macrophage‐specific markers iNOS, TNF‐α, ARG1, and CD163 to identify M1‐ (pro‐inflammatory or classically activated) or M2‐like (anti‐inflammatory or alternatively activated) macrophage phenotypes. The M1‐like polarization of macrophages is associated with inflammation and severity of SCI, while M2‐like macrophages reduce local inflammation and play neural promoting functions.^[^ ^36^ ^]^ It has been reported that the anti‐inflammation of H_2_S is through its ability to promote M2‐like (anti‐inflammatory) differentiation of macrophages.^[^ ^37^ ^]^ Cells cultured with OMSF@JK presented higher gene expression of M2‐like macrophages compared to cells cultured with LPS. Therefore, macrophage elongation is considered to be neatly related to M2‐like macrophage with anti‐inflammatory activities.^[^ ^38^ ^]^ Studies have shown that macrophage cells alternated morphological structure with the different stimuli of molecular factors.^[^ ^39^ ^]^ When stimulated with IFN‐γ or LPS, which are recognized for M1‐like polarization, macrophages display large and around the shape. However, M2‐like macrophage polarization is associated with an elongated morphology of the macrophages as observed in this work (Figure S7, Supporting Information). The morphological changes of macrophages had a link with their functional properties and it has been reported that the elongated stage tended to inhibit further M1‐like polarization.^[^ ^40^ ^]^

SEM data showed that PF‐OMSF@JK had a fine‐oriented tubular presence and reticular networks (Figure 2). This morphological structure is of utmost prominence as the oriented pores are eligible for supporting oxygen and nutrients to the cells encapsulated.^[^ ^41^ ^]^ Moreover, the crosslinking of the hydrogel produces morphologies that can decrease and increase the rate of permeation and diffusion therapeutic payloads.^[^ ^42^ ^]^ Previous studies have shown that slower degradation may be performed by mixing Pluronic F‐127 with different additives (e.g., polymers), which may be the case upon the addition of OMSF@JK to the hydrogel. These modifications may benefit the purpose of a specific graft, such as enhanced mechanical properties and prolonged H_2_S sustained release. From these measurements, it is evident that the storage and loss modulus of the developed composite hydrogel closely matches the modulus values of the spinal cord reported in the literature. In this regard, Bartlett et al. reported the storage modulus of different portions of the spinal cord to be between 3 and 11 kPa, while the loss modulus of the spinal cord varied from close to 1 to 4 kPa when measured between 1 and 50 Hz shear frequency.^[^ ^43^ ^]^ These mechanical properties of the presented thermoresponsive hydrogel (PF‐OMSF@JK) can be attributed to the phase reverse thermal gelation of the poloxamers under certain conditions. Specifically, when the poloxamer concentration and/or temperature of the poloxamer solution is higher than the critical micellization concentration, these polymers self‐assemble to form thermodynamically stable micelles.^[^ ^44^ ^]^ At an appropriate temperature, poloxamer micelles form a highly ordered and packed structure, which leads to the entanglement of the polymeric chains forming a gel.^[^ ^45^ ^]^

This data indicated that PF‐OMSF@JK could significantly prolong the releasing time of H_2_S and become more approved for clinical applications (Figure S9, Supporting Information). Thus, pH‐dependent release performance is a desired feature of the drug carrier systems, especially the ones intended for stem cell therapy for SCI because of the pH dynamic changes during the SCI healing process.^[^ ^46^ ^]^ Concisely, the low pH at the early stage of injury promots the fast release of H_2_S from PF‐OMSF@JK to promptly reduced the inflammation at the SCI site during this acute inflammatory stage. During the healing process, the pH progressively increases to the normal range which manages PF‐OMSF@JK to mild H_2_S release in a slow manner to extend prolonged restore SCI effects.

The DPSCs proliferation capacities when encapsulated in PF‐OMSF@JK2 hydrogel were evaluated in vitro (Figure S10, Supporting Information). These results correspond well with previous studies, which showed that PF‐127 without supporting molecular factors provided an appropriate MSCs environment such as DPSs to differentiate into osteogenic and neurogenic cells.^[^ ^47^ ^]^ The results acquired here are important for the transplantation of DPSCs‐based therapies as they can be quickly transplanted into the injury site with high viability retention as required for the SCI site. This unique feature makes PF‐OMSF@JK2/DPSCs favorable candidates for cell therapy applications.

Accumulated evidence shows that NF‐kB signaling pathways are particularly important for regulating neuroinflammation under a wide variety of circumstances.^[^ ^48^ ^]^ Both H_2_S donors NaHS (fast acting) and GYY4137 (slow acting) were found to decrease LPS‐induced activation of NF‐κB and release of TNF‐α and IL‐1β from macrophage.^[^ ^14^, ^49^ ^]^ Recent studies have implied the beneficial effects of DPSCs on neuroinflammation after acute SCI, related to decreased local inflammation (Figure 3).^[^ ^7a^ ^]^ Furthermore, the protective effects of DPSCs on functional recovery in contusive SCI rats have been established. The results verify that the SCI healing and functional recovery upon PF‐OMSF@JK/DPSCs treatment is associated with a decrease in local inflammation by stem cells and H_2_S by inhibiting NF‐kB signaling (Figures 3 and 4). This was confirmed by the protein expression of NF‐kB and other inflammatory markers using Western blotting and IHC analysis on spinal cord tissue of SCI rats treated with PF‐OMSF@JK/DPSc hydrogel. Results showed that the expression of IL‐6, TNF‐α, and NF‐kB was pretty high in the SCI group. After in suit injection of PF‐OMSF@JK/DPSC, this trend was significantly reversed. The reason for this might be that the sustained release of H_2_S (JK) from the hydrogel could invariably inhibit inflammation leading to continual repair of SCI.

With synergistic effects on nerve regeneration, Hydrogen sulfide (H_2_S) has been shown to participate in neuronal modulation and protection in mammals in recent years.^[^ ^50^ ^]^ Both PF‐OMSF/DPSCs and PF‐OMSF@JK2/DPSCs have demonstrated neuroprotective and regenerative capacities for sustaining neural growth and extension after prolonged denervation (Figure 5).^[^ ^7^, ^26^ ^]^ It is worth mentioning that the DPSCs can also strongly stimulate cells and factors to promote remodeling around the lesion region of the injured nerve, which can contribute to axon regrowth and repair by enhancing the transit of more nutrients and relieving toxic metabolites ^[^ ^6b^ ^]^. In the present study, we demonstrated that the co‐application of PF‐OMSF/DPSCs and PF‐OMSF@JK2/DPSCs loaded onto thermosensitive hydrogel led to a robust neuroprotective response with the extensive generation of new myelin en‐sheathing axons at SCI. Compared with the SCI group, the expression levels of MAP‐2 were significantly increased (Figure 5), reflecting microtubule stabilization and growth cone formation.

## 4 Conclusion

We have developed a thermo‐responsive hydrogel comprising MSNs with the ability to locally deliver H_2_S with DPSCs for promising SCI repair. The H_2_S donor‐delivering smart hydrogel was prepared by mixing OMSF nanoparticles and PF‐127 hydrogel matrix. The cross‐sectional morphologies of the PF‐OMSF@JK hydrogel exhibited a continuous and porous structure, which could facilitate the nutrient transfer, proliferation, and cell adhesion. The composite hydrogel prolonged the release of H_2_S, which enabled control of inflammation and proved advantageous for the differentiation of the incorporated DPSCs, eventually responsible for the formation of new tissue at the CI site. The work found an excellent SCI repair upon local application of PF‐OMSF@JK2/DPSCs, as a novel H_2_S‐releasing matrix could enhance nerve regeneration. This study unveiled the SCI treatment process achieved by this smart hydrogel with dual H_2_S release and DPSCs delivery is driven by control over the local SCI inflammation. We believe the presented PF‐OMSF@JK2 is a simple and robust local gas delivery system that holds strong potential for clinical translation and delivery of other gaseous therapies along with stem cells to treat SCI and other diseases.

## 5 Experimental Section

### Materials and Chemicals

Pluronic F‐127 (PF‐127), lithium 2‐(phenylphosphonothioylamino)−3‐phenylpropanoate (hydrogen sulfide donor) (JK), triethoxy(octyl)silane (OTS), hexadecyltrimethylammonium bromide (CTAB), LPS from *Escherichia coli*, and MAP‐2 antibody were obtained from the Sigma Aldrich (USA). Tetraethyl orthosilicate (TEOS) was obtained from Alfa Aesar (China). Paraformaldehyde (PFA) Triton X‐100 radioimmunoprecipitation buffer, *tris*‐HCl, zinc acetate, sodium hydroxide (NaOH), iron (III) chloride (FeCl_3_), *N*‐dimethyl‐phenylenediamine sulfate, and tetrahydrofuran (THF) were purchased from Aladdin Industrial Corporation (Shanghai, China). Fetal bovine serum (FBS), α‐modified Eagle's medium (MEM‐α), Eagle's minimal essential medium (DMEM), Neurobasal‐A medium supplemented B27, recombinant bFGF, recombinant epidermal growth factor (EGF), phosphate‐buffered saline (PBS), and penicillin‐streptomycin were bought from Gibco (Invitrogen, USA). Human antibody of CD90 was purchased from BD Pharmingen (USA), and human antibodies of CD14 and HLA‐DR were purchased BioLegend (USA). RNA extraction kit was obtained from Sangon Biotecholoy (Shanghai, China). PrimeScript RT kits and Reverse RNA kit were obtained from Takara Bio Inc. (Kyoto, Japan). TNF‐α antibody was purchased from Abcam. Antibodies of IκB‐α, NF‐κB, and acetyl‐α‐tubulin were purchased from Cell Signaling Technology (Danvers, USA). Interleukin 6 antibody (IL‐6) was obtained from Affbiotech (USA).

### Synthesis of MSN and OMSN

To prepare MSN, PF‐127 (5 mg) and CTAB (200 mg) were dispersed in 96 mL deionized (DI) water. The final pH of the solution was adjusted to 11.7 with NaOH. Then 1 mL of TEOS solution was added rapidly into the solution at 600 rpm for 90 min, where the reaction was carried out in a heated water bath (80 °C). To synthesize octyl‐MSN (OMSN), an octyl crosslinking on MSN was performed. Briefly, 0.25 mL of OTS was added to 10 mL of THF and was slowly added to the mixture after the above‐mentioned reaction. The mixture was stirred in water bath for 2 h (600 rpm, 80 °C). To collect the OMSN particles, the mixture solution was cooled down to room temperature and centrifuged four times (25 min per centrifuge, 9000 rpm, 20 °C) with DI water in the first three times and methanol in the last centrifuge. Then those CTAB‐MSN and CTAB‐OMSN were oven‐dried for 12 h at 55 °C.

To extract CTAB surfactants temptlet from MSN and OMSN, CTAB‐MSN and CTAB‐OMSN (0.5 g) were dissolved in 50 mL methanol with hydrochloric acid (2.5 mL) stirring (600 rpm, 24 h, 20 °C). Then, solutions were centrifuged for 25 min at room temperature (9000 rpm) and washed three times with DI water and twice with methanol. Finally, those MSN and OMSN were dried at 55 °C for 12 h.

### Preparation of JK‐Loaded OMSF (OMSF@JK)

To synthesize polymer PF‐127/JK coated OMSN, noted as OMSF@JK in this paper, the following procedures were carried out and briefly described below. First, JK (5 mg) was dissolved in 2 mL *tris*‐HCl solution (50 mm, pH 8.5) with 50 mg of OMSN and sonicated (80 w, water bath), then the suspension was stirred at 4 °C for 24 h. After that, 7 mL of polymer PF‐127 solution in water (5 mg mL^−1^) was added to the above mixture and vigorously stirred at 4 °C for another 12 h. OMSF@JK were collected at 9000 rpm for 25 min and redisposed in 20 mL of polymer PF‐127 solution in water (5 mg mL^−1^) at 4 °C. The above‐mentioned sonication and stirring steps were repeated after centrifugation. Finally, OMSF@JK was precipitated and washed with DI water twice to remove residual PF‐127. In this paper, the OMSF@JK was prepared with different concentrations of JK: 30 µg of JK noted as OMSF@JK1, 60 µg of JK noted as OMSF@JK2, and 100 µg of JK noted as OMSF@JK3.

### Characterization of Nanoparticles

The morphologic features of MSN were scanned by transmission electron microscopy (TEM, Hitachi, Japan) at Wenzhou Institute of Biomaterials & Engineering, where the size of modified nanoparticles was measured and compared. The nanoparticles’ composition was assessed by an FTIR (Vertex 70, Bruker) at Wenzhou Institute of Biomaterials & Engineering. Also, the thermal gravimetric of the nanoparticles and thermal stability and the fraction of unstable components in nanoparticles were determined by using thermogravimetric analysis (TGA) in the temperature range of 50–600 °C under nitrogen atmosphere. The pore structure and surface area of the nanoparticles were determined by the nitrogen adsorption‐desorption measurements at 77.300 K using a Micromeritics Autochem ASAP 2020 V3.00H unit at the Wenzhou Institute of Biomaterials & Engineering.

### Isolation, Culture, and Identification of DPSCs

The extraction, isolation, and culture of DPSCs were carried out based on a previous study.^[^ ^51^ ^]^ The use of DPSCs designated in this paper was approved and revised by the Ethics Committee of Wenzhou Medical University at Hospital and School and of Stomatology (No. WYKQ2018008SC). Briefly, permanent teeth were gathered from the Maxillofacial Surgery Department, Hospital of Stomatology at Wenzhou Medical University. Pulp tissues were extracted, minced into small pieces, digested, cultured in MEM‐α supplemented with 20% FBS, 100 U mL^−1^ penicillin, and 100 µg mL^−1^ streptomycin and incubated at 37 °C incubator (5% CO_2_). The medium was replaced on day 5 and then every 2 days. DPSCs morphology was observed and photographed under a light microscope. The stemness properties of DPSCs were identified by using flow cytometry with the antibodies of human HLA‐DR CD90, and CD14. The results were analyzed by using CytoFLEX flow Imaging cytometers (Beckman Coulter, California, USA).

### Cytotoxicity on DPSCs

The viability of OMSN against DPSCs was evaluated by a CCK‐8 assay. Cells were seeded in 48‐well plates (1 × 10^4^ cells per well) and cultured in MEM‐α supplemented with 10% FBS and 100 U mL^−1^ penicillin, and 100 µg mL^−1^ streptomycin for 24 h at 37 °C. After the DPSCs attachment, the medium was removed and the DPSCs were incubated in 400 µL well^−1^ of fresh MEM‐α containing 0, 12.5, 25, 50, 75, and 100 µg of OMSN and OMSF for 24 h at 37 °C. To investigate the DPSCs viability, 200 µL of MEM‐α and 20 µL of CCK‐8 were added to each well and incubated for 2 h at 37 °C. The cellular viability of JK and OMSF@JK against DPSCs was assessed by a Cell Counting Kit‐8 (CCK‐8). After culturing, the culture medium was removed and the cells were incubated in 400 µL of fresh MEM‐α containing (0, 3, 5, 15, 30, 40, 60, and 100 µg mL^−1^) of JK and (0, 3, 5, 15, 30, 40, 60, and 100 µg mL^−1^) of OMSF@JK in the incubator at 37 °C on days 1 and 3. Finally, 100 µL of the solution from each well was transferred to a 96‐well plate and used to measure the absorbance at 450 nm in a plate reader.

### Neurogenic Differentiation of DPSCs and Culture of d‐DPSCs

The neurogenic differentiation of DPSCs was performed similarly as in an earlier work.^[^ ^52^ ^]^ Briefly, DPSCs were cultured in 48‐well plates at 1 × 10^3^ cells well^−1^ in complete α‐MEM containing 10% FBS 100 U mL^−1^ penicillin, and 100 µg mL^−1^ streptomycin and incubated for 24 h. Then neurogenic induction medium (NM), Neurobasal‐A medium supplemented with 2% B27, 20 ng mL^−1^ EGF, and 20 ng mL^−1^ bFGF, was applied. Cells that were under neurogenic induction were challenged with LPS (100 ng mL^−1^) in absence or presence of nanoparticles (30 µg of JK noted as OMSF@JK1, 60 µg of JK noted as OMSF@JK2, and 100 µg of JK noted as OMSF@JK3) for 3, 6, and 12 days. DPSCs cultured with 400 µL medium were noted as the control group, and DPSCs cultured with NM were noted as the NM group. At designated timepoints, DPSCs were first photographed for proliferation and morphology comparison under a light microscope and then fixed for immunocytochemistry analysis with primary antibodies: CD 146 and anti‐GFAP at day 3 and MAP2 and anti‐GFAP at days 6 and 12. Cell nuclei were counterstained with DAPI.

### Expression of Neurogenic Genes

DPSCs were seeded in 6‐well plates at a density of 1 × 10^5^ cells well^−1^ and were cultured, neurogenically inducted, and challenged with LPS and OMSF@JK at varied concentrations for 12 days. Total RNA was extracted using a Sangon kit according to the manufacturer's protocol. Then 2 µg of extracted RNA was reversely transcribed to cDNA using a cDNA Takara Reverse Kit. The gene expressions of Fibronectin, MAP2, Nestin, and NeuroD1 were calculated with the corresponding primers (Table S1, Supporting Information).

### RAW264.7 Cell Culture and Pro‐Inflammatory Expression

RAW 264.7 cell line was received as a kind gift from the Wenzhou Institute of Biomaterials and Engineering. The cells were seeded in 24‐well plates at 1 × 10^5^ cells cm^−2^ and cultured with DMEM with 10% FBS and 1% penicillin‐streptomycin for 24 h. Then medium was replaced by fresh medium supplemented with LPS, LPS+OMSF@JK1, LPS+OMSF@JK2, and LPS+OMSF@JK3 for 24 h. Then, the cells were fixed (2.5% glutaraldehyde, 30 min), dehydrated (gradient methanol), and observed by SEM. Also, these RAW 264.7 cells were fixed (4% PFA), washed (PBS 5 min, three times), permeabilized (0.1% Triton X‐100, 5 min and 5% BSA, 30 min) at 37 °C. The antibody of IL‐6 as pro‐inflammatory factor was stained by immunofluorescent label. The nuclei were counter‐stained with DAPI. Images were taken with a fluorescence microscope.

### Expression of Pro‐Inflammatory Genes

To evaluate the expression of M1‐associated cell surface markers such as iNos and TNF‐α and markers specific to M2‐like macrophages such as ARG1 and CD 163 on the RAW cells cultured in 3.5.1. cDNA was transcribed from RNA by using a Prime Script RT kit. Quantitative PCR samples were performed with a total volume of 20 µL with identical primers (Table S1, Supporting Information) and a PCR SYBR Green Kit. Data were normalized with GAPDH and analyzed using the 2^−ΔΔCt^ method.

### Preparation of Hydrogel

The hydrogel was prepared by the cold method.^[^ ^7b^ ^]^ where 17% of PF‐127 polymer was slowly added to PBS (pH 7.4) with or without OMSF@JK in water bath at 4 °C and then vigorously stirred for 12 h at 4 °C.

### Characterization of PF‐OMSF@JK Hydrogel

The cross‐sectional micromorphology of the PF‐OMSF@JK hydrogel was examined using SEM. Hydrogels were frozen dried at −80 °C for 48 h, and cut into thin slices using scalpel blades. The mechanical, gelation characteristics, and rheological properties of hydrogels were tested by a hybrid TA rheometer. The amplitude sweep and transition temperature were studied using the sol‐gel method, where parallel stainless‐steel plates (25 mm) were applied on the hydrogels, and the shear strain was placed to 1%, the shear frequency was set at 10 rad s^−1^.

### H_2_S Release from PF‐OMSF@JK Hydrogel

To study the pH dual responsiveness of PF‐OMSF@JK hydrogel, the release of H_2_S from the hydrogel was measured at pH 6.0 and 7.4. PF‐JK and PF‐OMSF@JK2 (both containing equivalent 2 mg JK and 17% of PF‐127) were placed in 10 mL PBS at pH 6.0 and pH 7.4 respectively. Every 5 h, 0.2 mL solution was retrieved and added with 50 µL zinc acetate (1% w/v in H_2_O) and 6.25 µL NaOH (1.5 m). Then the mixture solution was centrifuged (12 000 rpm, 1 h, 20 °C), and the pellet was dissolved in 100 µL FeCl_3_ (30 mm in 1.2 m HCl) and 100 µL *N*,*N*‐dimethyl‐phenylenediamine sulfate (20 mm in 7.2 m HCl). Finally, 0.5 mL water was added and transferred 200 µL to a 96‐well plate, settled for 5 min, and read at 670 nm using an optical density microplate reader. The release was observed for a total of 65 h.

### 3D DPSCs Encapsulation

To perfuse the hydrogel with cell culture medium, 170 mg mL^−1^ PF‐127 and 60 µg mL^−1^ OMSF@JK2 were dissolved in 30 mL α‐MEM (supplemented with 1% mixture of penicillin G and streptomycin and 10% FBS) under moderate stirring (600 rpm) at 4 °C for 12 h. Then, this hydrogel perfused medium was sterilized under UV light for 1 h in an ice bath. To study the viability of DPSCs cultured with this PF‐127 and PF‐OSMF@JK2 hydrogel, DPSCs (1 × 10^6^ cells mL^−1^) were slowly mixed with hydrogel in the ice bath. Then, 200 µL well^−1^ DPSCs infused hydrogel was placed in 48‐well plate and incubated at 37 °C for 20 min to allow gelation. 200 µL well^−1^ pre‐warmed α‐MEM was gently added to prevent dehydration.

To investigate the DPSCs viability cultured for 1 and 3 days inside the hydrogels, 200 µL well^−1^ of MEM‐α and 20 µL well^−1^ of CCK‐8 were added and incubated for 2 h at 37 °C. 100 µL well^−1^ incubated solution was transferred to a 96‐well plate and read at 450 in a plate reader.

To visualize 3D of DPSCs after 1 day of culture inside the hydrogels, cells were washed with PBS for three times, fixed with 4% PFA for 15 min, and rinsed with PBS for three times. DPSCs were permeabilized by 0.1% Triton X‐100 in PBS for 5 min and 5% BSA for 30 min at 37 °C. Then DPSCs were labeled by 1 mg mL^−1^ of phalloidin‐TRITC and 2 mg mL^−1^ DAPI. Samples were photographed by a fluorescence microscope (Eclipse 80i, Nikon, Japan).

### SCI Model and Treatment

To evaluate the effect of PF‐OMSF@JK/DPSCs hydrogel in SCI‐injured rats, 40 adult female Sprague−Dawley rats (210−260 g) were obtained from Animal House of Chinese Academy of Sciences (Shanghai, China). All animal‐related work was prepared according to the Guide of Chinese National Institutes of Health and approved by the Animal Care and Use Committee of Wenzhou medical University (no. WYKQ‐2018‐008SC). Rats were acclimated at the animal center for 12 days and supplied with free access to food and water. They were randomly divided into four groups: PF‐OMSF@JK/DPSCs, PF‐OMSF/DPSC, SCI, and control groups. SCI injury was established as previously described.^[^ ^7a^ ^]^ The animals were anesthetized by intraperitoneal injection of 8% chloralhydrate (2.9 mL kg^−1^). Rats were fixed on a cork plate in a prone position. The surgical area was shaved, disinfected, and dressed. A 2 cm long vertical incision was made along the midline from T8 to T10 of the spine to uncover the vertebral columns. T9 vertebrae of spine were identified, and the vertebral bone of spine was removed to expose spinal cord. A moderate injury was performed by clapping a vascular clip on spinal cord (30 g, 2 min). Then, hydrogels (100 µL) were applied using a micro‐syringe at the injured site. SCI group received 100 µL of sterile saline. Animals in the control group went through the procedures without injury. Postsurgical care was provided including infection control, pain management, hydration and dietary care, individual housing, etc. In this regard, subcutaneous (s.c.) injections of marbofloxacine at 5 mg k^−1^ g dose were given immediately after surgery and on the 2nd and 4th day after the surgery to prevent any infection. The urine in the bladder was monitored for presence of blood. For the analgesic, s.c. buprenorphine injections of 0.05 mg k^−1^ g were given to the animals twice a day for the first 3 days after the surgery. The rats were manually urinated three times a day till auto‐urination was recovered. Animals were observed for 7 days.

At the end of the 7‐day period, the rats were anesthetized using a 100 mg k^−1^ g dose of sodium pentobarbital. Once the rats reached deep anesthesia (assessed by toe‐pinch response method), they were placed on the shallow tray. Then a small lateral incision of about 5 to 6 cm was made just below the rib cage through the abdominal wall. After carefully separating the liver from the diaphragm, an incision in the diaphragm was made parallel to the entire length of the rib cage to reveal the pleural cavity. The sternum was carefully lifted away and the tissue connected to the heart was trimmed. This provided a clear view of the heart and the major vessels. After that a small incision was made at the posterior end of the left ventricle and a blunt 15‐gauge blunt‐ needle was passed into the ascending aorta through the cut in the ventricle. Note, the tip of the needle was visible through the wall of the aorta, and hemostatic clamps were used to prevent any leakage. Next, an incision to the right atrium of the rats was made. Through this incision, normal saline (0.9%) was first injected until the liver became clear and then the buffered PFA solution was perfused until the rats became stiff. The effluent PFA solution was collected for disposal according to the institutional safety regulations at the Wuhan Medical University (WMU). Then, a 5 cm long spinal cord segment with the epicenter in the middle was excised and fixed with 4% PFA for 24 h.

### Western Blot

The spinal cord tissue at the T9 level was collected and lysed in radioimmunoprecipitation buffer with protease inhibitor cocktail for 30 min in the ice bath and centrifuged at (12, 000 rpm, 4 °C) to get the supernatants. The protein concentration was measured by a high‐sensitivity Bradford protein assay. Protein (80 µg) was prepared by electrophoresis in 10% SDS‐PAGE gels and then transferred into poly(vinylidene difluoride) (PVDF) membrane. 5% blocked milk in 0.05% *tris* buffered saline −0.05% Tween was blocked nonbinding protein for 2 h and incubated with first antibodies at 4 °C for 16 h: IL‐6, TNF‐α, IκB‐α NF‐κB, and MAP‐2. Acetyl‐α‐tubulin, and then samples were preserved with the second antibodies (1 h, room temperature). The protein exposure was analyzed by the using ChemiDoc XRS imaging system (Bio‐Rad Laboratories, Hercules, CA). The experiments were done in triplicate.

### Immunohistochemistry

Immunohistochemistry evaluation of T9 of spinal cord level was made on day 7. The length of the tissue was around 5 cm and it was fixed with paraformaldehyde (4%, pH = 7.4) at room temperature for 24 h and stored in the refrigerator until required for further analysis. To dehydrate the spinal cord sections, the samples were rinsed with copious amounts of water followed by incubation in different alcohol concentrations (50%, 70%, 80%, and 95%) for 45 min each. At the end, the sections were placed in 100% alcohol for 1 h. The tissue sections were cleared in xylene for 2 h, requiring xylene refreshment at every hour. Next, the tissue sections were immersed in paraffin for 3 h to form a tissue section embedded block of paraffin. The paraffin embedded tissue was cut to achieve sections of 5 µm thickness, which were collected in a water bath at 40 °C. The Tissue sections were then collected onto glass slides for immunohistochemical labeling. The slides were deparaffinized by placing them in xylene for 2 h, requiring xylene refreshment every hour. After deparaffinization, the slides were placed into 100% alcohol for 9 min with alcohol refreshed every 3 min. The slides were placed in different alcohol solutions (95%, 70%, and 50% alcohol) for 3 min each followed by blocking of the endogenous peroxidase activity in the sections by incubating them in 3% H_2_O_2_ solution at room temperature for 10 min. Slides were then dipped in 400 mL of PBS for 10 min to rinse followed by a dip in 10 mm citrate buffer at pH 6.0 for sterilization. After cooling, the slides were washed in three changes of PBS for 5 min each time. Next, 100 µL 10% FBS in PBS was added to the slides as a blocking buffer and incubated in a humidified chamber at room temperature for 1 h. After that period, the slides were rinsed with PBS and immunohistochemical labeling with target antibody was carried out in 0.5% of bovine serum albumin solution in PBS in a humidified chamber at 37 °C for 1 h. The slides were washed with PBS for 10 min prior to the application of the next immunohistochemical labeling step, which was carried out in a manner similar to the first labeling step. Note, for this work, the samples were treated by first antibodies of IL‐6, NF‐κB, and IκB‐α, followed by their corresponding secondary antibodies. After antibody labeling, the slides were washed in PBS for 10 min followed by incubation with 100 µL of diluted Sav‐HRP conjugates in a humidified chamber at 37 °C for 30 min. Next, the slides were washed in PBS for 10 min followed by the application of 100 µL of 3,3′‐Diaminobenzidine (DAB) solution on the slides to develop the color due to antibody staining. The slides were incubated with DAB for up to 5 min and washed with PBS and milli‐Q water for fifteen min. The tissue sections were dehydrated again through four changes of 95%, 95%, 100%, and 100% of alcohol for 5 min each time. Before being sealed with coverslips, the samples were hematoxylin and eosin (H&E) stained for 8 min. The samples were observed by a light microscope (Nikon, TS100, and Japan).

### Statistical Analysis

Data were presented as means ± standard deviations. Statistical comparisons were carried out using one‐way ANOVA tests. Statistical significance was defined as *p < 0.05, **p < 0.01.

### Ethics Approval and Consent to Participate

The use of DPSCs designated in this paper was approved and revised by the Ethics Committee of Wenzhou Medical University at Hospital and School and of Stomatology (No. WYKQ2018008SC). Written consent was acquired from all donating individuals. All animal‐related work was prepared according to the Guide of Chinese National Institutes of Health and approved by the Animal Care and Use Committee of Wenzhou Medical University.

## Conflict of Interest

The authors declare no conflict of interest.

## Author Contributions

A.A.A., Y.H., Y.L., and X.D. contributed equally to this work. A.A.A., Y.H., and Q.Y. conceived the study. J.A., D.F., Y.X., S.L., X.D. L.L., and X.Z. performed the experiments. A.A.A., Y.H., X.Z., T.K., and Q.Y. analyzed the data and results, and A.A.A., Y.P., and T.K. drafted the manuscript. Y.H., D.F., L.L., T.K., and Q.Y. revised the manuscript and approved the submission. All authors approved the final manuscript.

## Supporting information

Supporting Information

## Data Availability

The data that support the findings of this study are available from the corresponding author upon reasonable request.
