# Supplementary material for: Local Spinal Cord Injury Treatment Using a Dental Pulp Stem Cell Encapsulated H2S Releasing Multifunctional Injectable Hydrogel
Source: Adv Healthc Mater. 2023 Dec 16;13(9):2302286. doi: 10.1002/adhm.202302286 (PMC11469045; doi:10.1002/adhm.202302286)
Supplement: Supplementary file 1 — Supporting Information [file ADHM-13-2302286-s001.pdf]

ADVANCED HEALTHCARE MATERIALS

Supporting Information

for *Adv. Healthcare Mater.*, DOI 10.1002/adhm.202302286

Local Spinal Cord Injury Treatment Using a Dental Pulp Stem Cell Encapsulated H_2S Releasing Multifunctional Injectable Hydrogel

Abdullkhaleg Ali Albashari, Yan He, Yu Luo, Xingxiang Duan, Jihea Ali, Mingchang Li, Dehao Fu, Yangfan Xiang, Youjian Peng, Song Li, Lihua Luo, Xingjie Zan, Tushar Kumeria and Qingsong Ye PhD, DDS**

Supporting Information

Local Spinal Cord Injury Treatment using a Dental Pulp Stem Cell Encapsulated H_2S Releasing Multifunctional Injectable Hydrogel

Abdullkhaleg Ali Albashari[1,2†,], Yan He[3,4†], Yu Luo[1†], Xingxiang Duan[1†], Jihea Ali[5], Mingchang Li[6], Dehao Fu[7], Yangfan Xiang[2], Youjian Peng[1], Song Li[2], Lihua Luo[2], Xingjie Zan[8], Tushar Kumeria[9,10*], Qingsong Ye[1,2,4*]

[1] Center of Regenerative Medicine, Renmin Hospital of Wuhan University, Wuhan, Hubei, 430060, China
[2] School and Hospital of Stomatology, Wenzhou Medical University, Wenzhou, Zhejiang, 325035, China
[3] Laboratory for Regenerative Medicine, Tianyou Hospital, Wuhan University of Science and Technology, Wuhan, Hubei, 430064, China
[4] Oral Maxillofacial Department, Massachusetts General Hospital, Harvard Medical School, Boston, MA 02114, USA
[5] College of Life and Environmental Science, Wenzhou University, Wenzhou, Zhejiang, 325035, China
[6] Department of Neurosurgery, Renmin Hospital of Wuhan University, Wuhan, Hubei, 430060, China
[7] The First Renmin Hospital of Shanghai, Tongji University, Shanghai, 200940, China
[8] Wenzhou Institute, University of China Academy of Science, Wenzhou, Zhejiang, 325024
[9] School of Materials Science and Engineering, University of New South Wales, Sydney, NSW 2052, Australia
[10] Australian Center for NanoMedicine, University of New South Wales, Sydney, NSW 2052, Australia
† Contributed equally to this work

*Correspondence authors: Qingsong Ye: qingsongye@hotmail.com; Tushar Kumeria (t.kumeria@unsw.edu.au)

S1. Supporting Methods

S1.1. Expression of neurogenic genes and inflammatory genes

Some of the key neurogenic genes along with their primer sequences are listed in **Table S1**.

Table S1: Human neurogenic and rat inflammatory genes alongside their corresponding primer sequence for RT-PCR analysis.

Human Neurogenic Gens	Primer Sequence

MAP 2-F	AAC CCT TTG AGA ACA CGA CA
MAP 2-R	TCT TTC CGT TCA TCT GCC A
Nestin-F	CTG GAG CAG GAG AAA CAG G
Nestin-R	TGG GAG CAA AGA TCC AAG AC
Fibronectin-F	CAG TGG GAG ACC TCG AGA AG
Fibronectin-R	CAA AGA CTA CAA GGC TCC CT
NeuroD1-F	CCA CGG ATC AAT CTT CTC AG
NeuroD1-R	CAT GAT GTG AAT GGC TAT CG
Gapdh-F	ATG GGC AGC CGT TAG GAA AG
Gapdh-R	GAT CTC GCT CCT GGAA GAT GG
Rat Inflammatory Genes	Primer Sequence
TNF-α-F	CCA GGC AGG TTC TGT CCC TT
TNF-α-R	ATA GGC ACC GCC TGG AGT TC
iNos-F	GTT CTC AGC CCA ACA ATA CAA GA
iNos-R	GTG GAC GGG TCG ATG TCA C
ARG1-F	CAG AAG AAT GGA AGA GTC AG
ARG1-R	CAG ATA TGC AGG GAG TCA CC
CD163-F	TCA GCG ACT TAC AGT TTC CTC
CD163-R	GCC TTT GAA TCC ATC TCT TG
Gapdh-F	GGA TGC AGG GAT GAT GTT C
Gapdh-R	TGC ACC ACC AAC TGC TTA G

S2. Supporting Results

S2.1. Physical and Chemical Characterization of particles

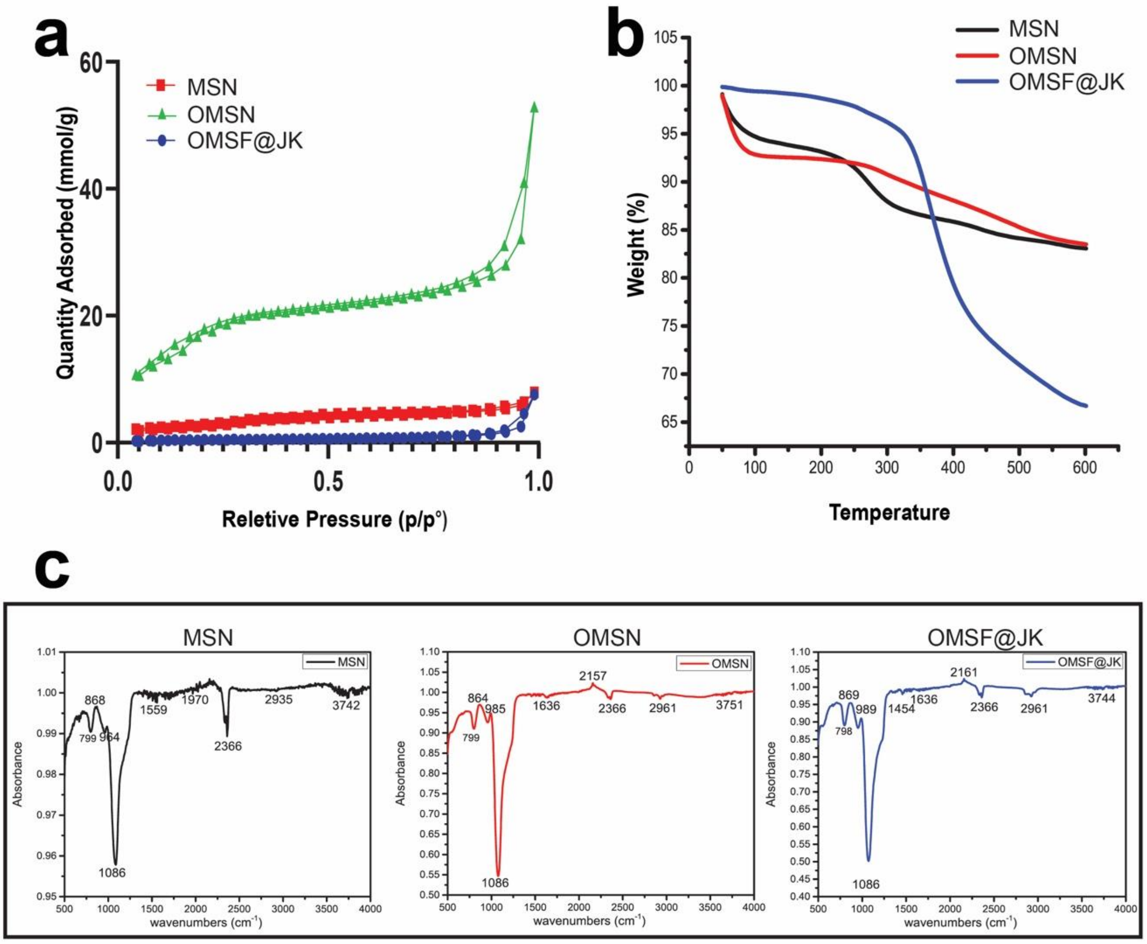

Fig. S1: Characterization of nanoparticles: (A) Nitrogen adsorption, OMSF@JK decreased N2 amount adsorbed level more than MSN and OMSN due to loading of JK and coating of polymer PF, which filled up and blocked the highly porous structure of OMSN. OMSN is much more porous than MSN, suggesting octyl-MSN is an efficient method to functionalize MSN for drug loading purposes. (B) Thermogravimetric analysis of MSN, OMSN, and OMSF@JK nanoparticles at 600 °C. The weight loss of blank MSN, OMSN, and OMSF@JK was 83.072%, 83.494%, and 66.691%. This result indicated: 1. OMSN is as stable as MSN till at 600 degrees despite OMSN being more porous. 2. OMSF@JK was synthesized from OMSN with the addition of JK and polymer PF. Being baked till 600 degrees, starting from 300 degrees, JK and polymer PF started to lose from OMSF-JK. There was only 65% weight left. This meant OMSN could load quite a lot, about half of its own weight in this case. (C) FTIR spectra of MSN, OMSN, and OMSF@JK. OMSF@JK exhibits a characteristic peak of CH weak stretch (3744 cm^{-1}), CH_3 medium stretch (2961cm^{-1}), C=C medium stretch scissor (2366 cm^{-1}), C=C

medium stretch anti symmetric bending (2161 cm^{-1}), C=O weak stretch (1636 cm^{-1}), C-O medium stretch (1454 cm^{-1}), C-H strong stretch (1086 cm^{-1}), and C-H medium stretch (798 cm^{-1}).

S2.2. Culture, morphological, and characteristics of DPSCs

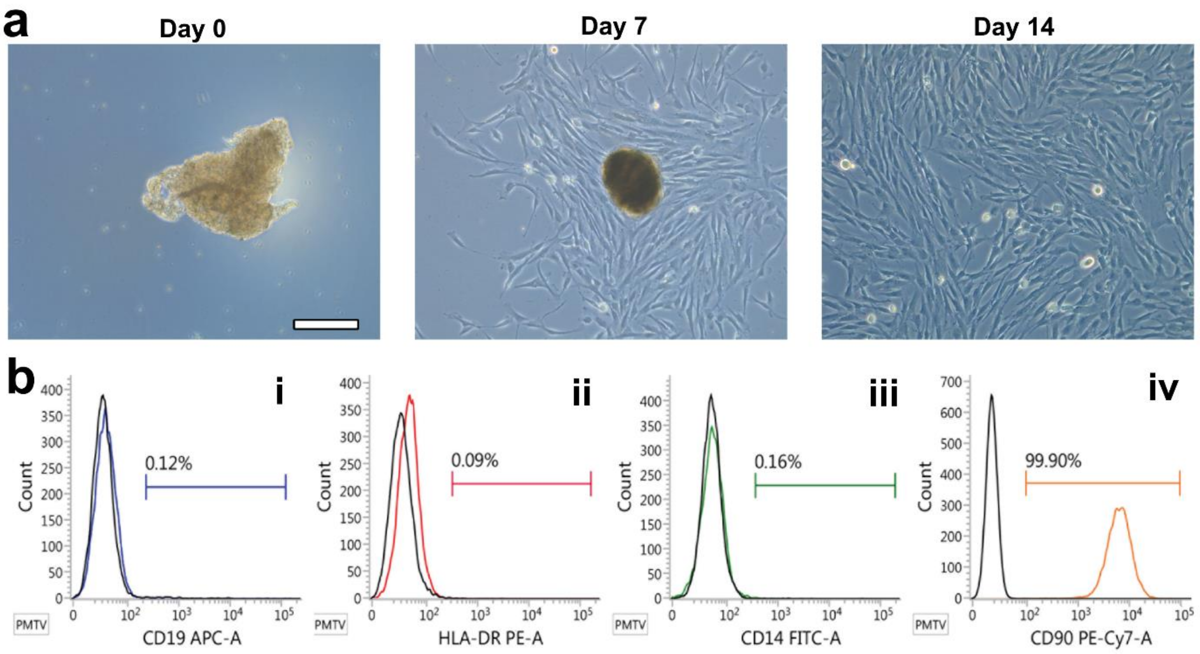

Fig. S2: (A) Human dental pulp tissue in culture dish on day 0 (left). Fibroblast-like cells emerged out around dental pulp tissue 7 days after pulp tissue was placed in the culture dish (middle). On day 14 of DPSCs reached 100% confluency in the culture dish (right). (B) The flow cytometry of surface marker expression of DPSCs. The results indicated that DPSCs negatively expressed the hematopoietic surface antigens CD19, HLA-DR, and CD14 and positively expressed MSC-like phenotypic marker CD90. Scale bar: 200 µm.

S2.3. Cytocompatibility of nanoparticles with DPSCs

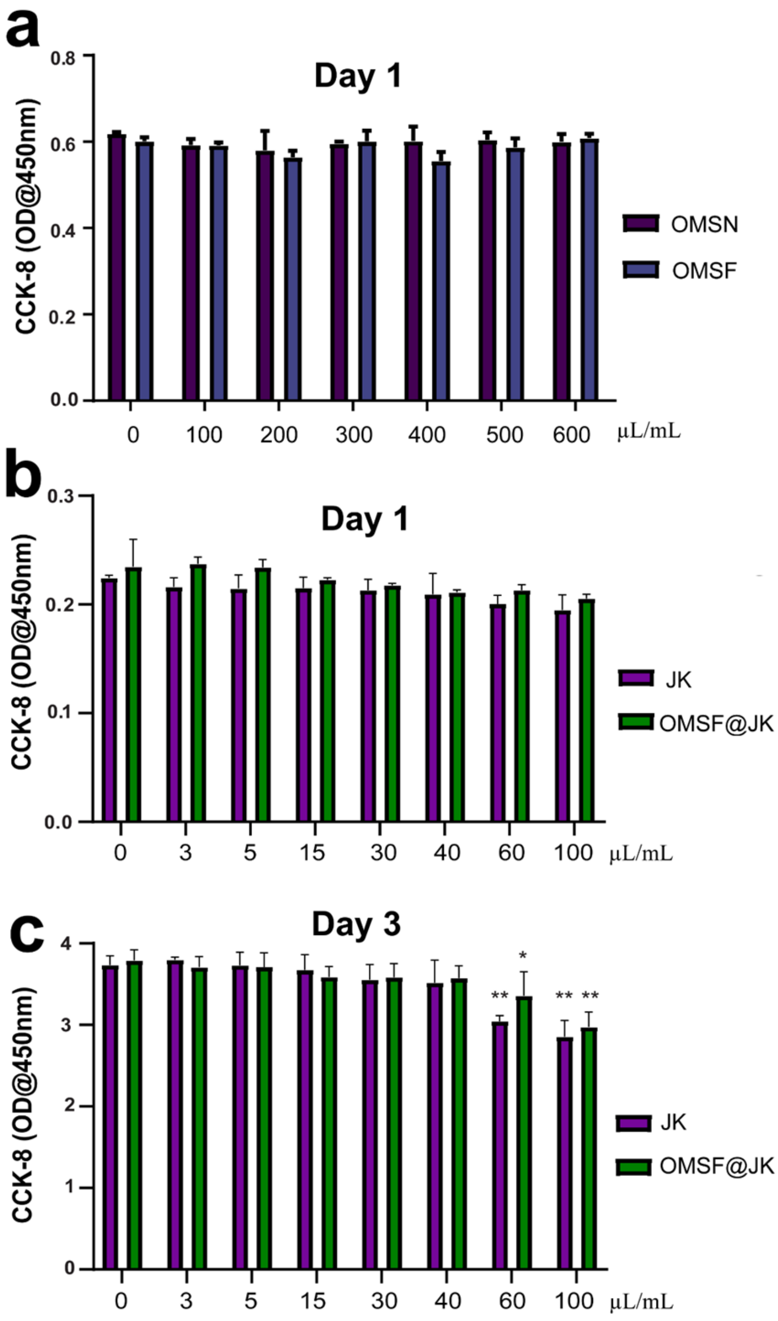

Fig. S3: Cytocompatibility of nanoparticles assessed on DPSCs: (A) DPSCs were cultured with OMSN and OMSF at different concentrations for 24 h. There was no statistical difference of cell viability exposed to nanoparticles at various dosages up to 600 μg/mL. DPSCs were cultured with the presence of JK and OMSF@JK for (B) 1 day and (C) 3 days. There was a non-significant decrease in cell viability at day 1 for the higher concentrations (>60 μg/mL). Whereas, the reduction in viability for the same concentrations on day 3 is clearly evident for both JK and OMSF@JK. Cell viability was assessed by CCK-8 assay at 450 nm. Data are shown as mean ± SD $*p < 0.05$, $**p < 0.01$.

S2.4. DPSCs morphology during neurogenic induction

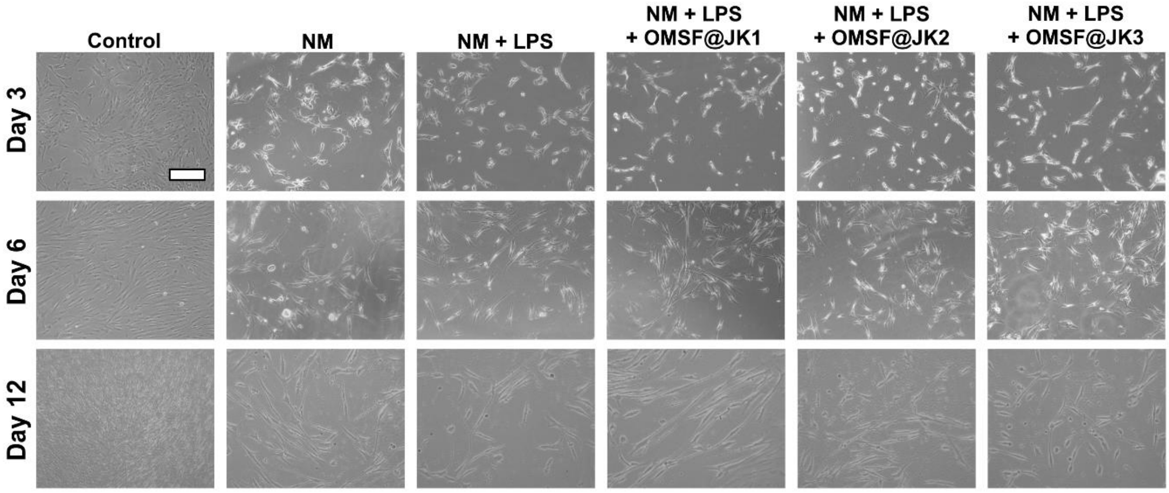

Fig. S4: Comparison of DPSCs morphology changed during neurogenic induction with and without the presence of LPS (100 ng/ml concentration) and nanoparticles OMSF@JK at various concentrations for 3, 6, and 12 days. When cultured in 400 µL/mL medium, the control, DPSCs kept the spindle shape and reached 100% confluency till day 12. When cultured in a neurogenic induction medium, the NM group, during 12 days period, cells gradually elongated and branched. When LPS was presented, the NM+LPS group, more round shape cells were seen on day 3 and some cells did not extend cellular processes on day 12. In three NM+LPS+OMSF@JK groups, on day 3, most DPSCs started to present a more spherical shape and advanced extending cells processes. On day 6, morphological characters typical of neurons were observed. Till day 12, most cells showed multi-or bipolar form like neurons. Scale bar: 200 µm.

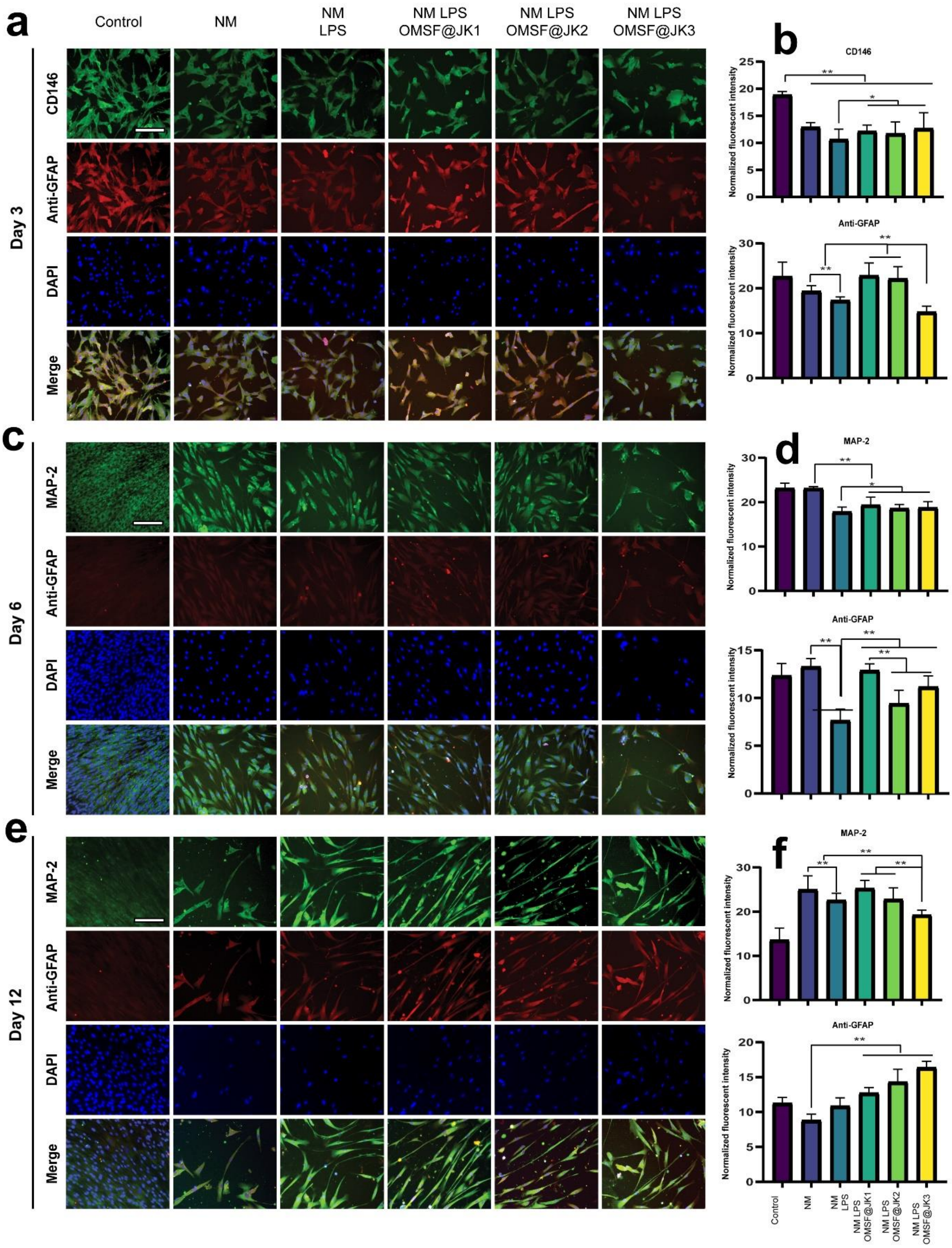

Fig. S5: The impact of LPS with and without OMSF@JK nanoparticles at various concentrations on the neurogenic differentiation of DPSCs, unveiled by the immunofluorescence staining and intensity quantification of surface markers: (A) Expression of CD 146 (green), a stemness marker, anti-

GFAP(red) neurons, and oligodendrocytes marker were chosen to stain the DPSCs cultured in nanoparticles for 3 days. The positive expression of CD146 presented the stemness of the DPSCs. Cellular nuclei were visualized with 4′, 6- diamidino-2-phenylindole (DAPI) (blue). (B) After 3 day of culture in a neurogenic medium (NM) with and without LPS and OMSF@JK, the stemness marker CD146 of the cells was significantly reduced compared with that of the control group ($p<0.01$). Cells in the NM+LPS group showed the lowest expression of neuronal marker anti-GFAP. Whereas with the addition of high and medium dosages of OMSF@JK, in NM+LPS+OMSF@JK1 and NM+LPS+OMSF@JK2 groups, the expression of anti-GFAP was significantly higher than that of the NM group ($p<0.01$). (C) Expression of MAP2 (green), a neural marker, and anti-GFAP (red) was chosen to stain the DPSCs cultured in nanoparticles for 6 days. (D) On day 6, the NM+LPS group showed the lowest neuronal surface marker expression among all groups. Compared with the NM+LPS group, the MAP-2 expression in all three NM+LPS+OMSF@JK groups was significantly higher ($p<0.05$), and the anti-GFAP expression in NM+LPS+OMSF@JK groups was significantly higher compared with NM LPS ($p<0.01$) E) Expression of MAP2 (green), anti-GFAP(red) were chosen to stain the DPSCs cultured in nanoparticles for 12 days. (F) On day 12, the MAP-2 expression of NM+LPS+OMSF@JK1 group was at a similar level as the NM group, and the anti-GFAP expression of all three NM+LPS+OMSF@JK groups was significantly higher than that of the control group ($p<0.01$), with the NM+LPS+OMSF@JK3 group being the highest. *$p < 0.05$, **$p < 0.01$. Scale bar: 100 µm.

S2.5. Neurogenic gene expression of DPSCs

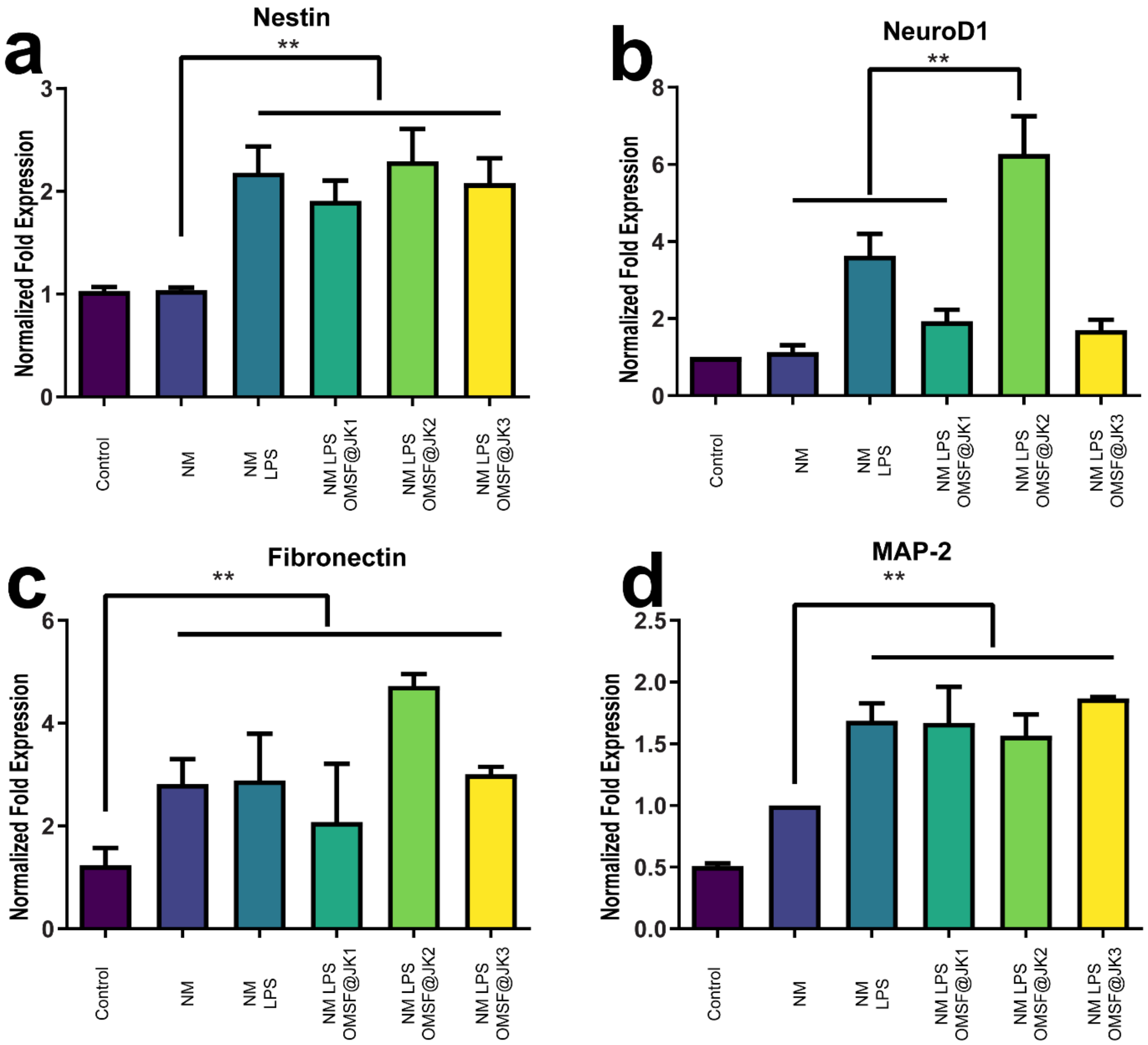

Fig. S6: Neurogenic gene expression of DPSCs treated by neuro-induction medium with and without LPS and OMSF@JK at varying concentrations for 12 days. To evaluate the changes in DPSCs intermediate gene expression, we performed real-time -PCR, whereas mRNA expression was assessed by real-time PCR. In data, neuronal markers like Nestin, NeuroD1, Fibronectin, and MAP-2 were expressed very well in OMSF/JK exposed group. The expression of each gene was normalized to that of GAPDH and reported as fold-changes. We found that OMSF/JK significantly increased the gene expression of Nestin, NeuroD1, Fibronectin, and MAP-2. $*p < 0.05$, $**p < 0.01$

S2.6. Anti-inflammatory Effect of nanoparticles on Macrophages

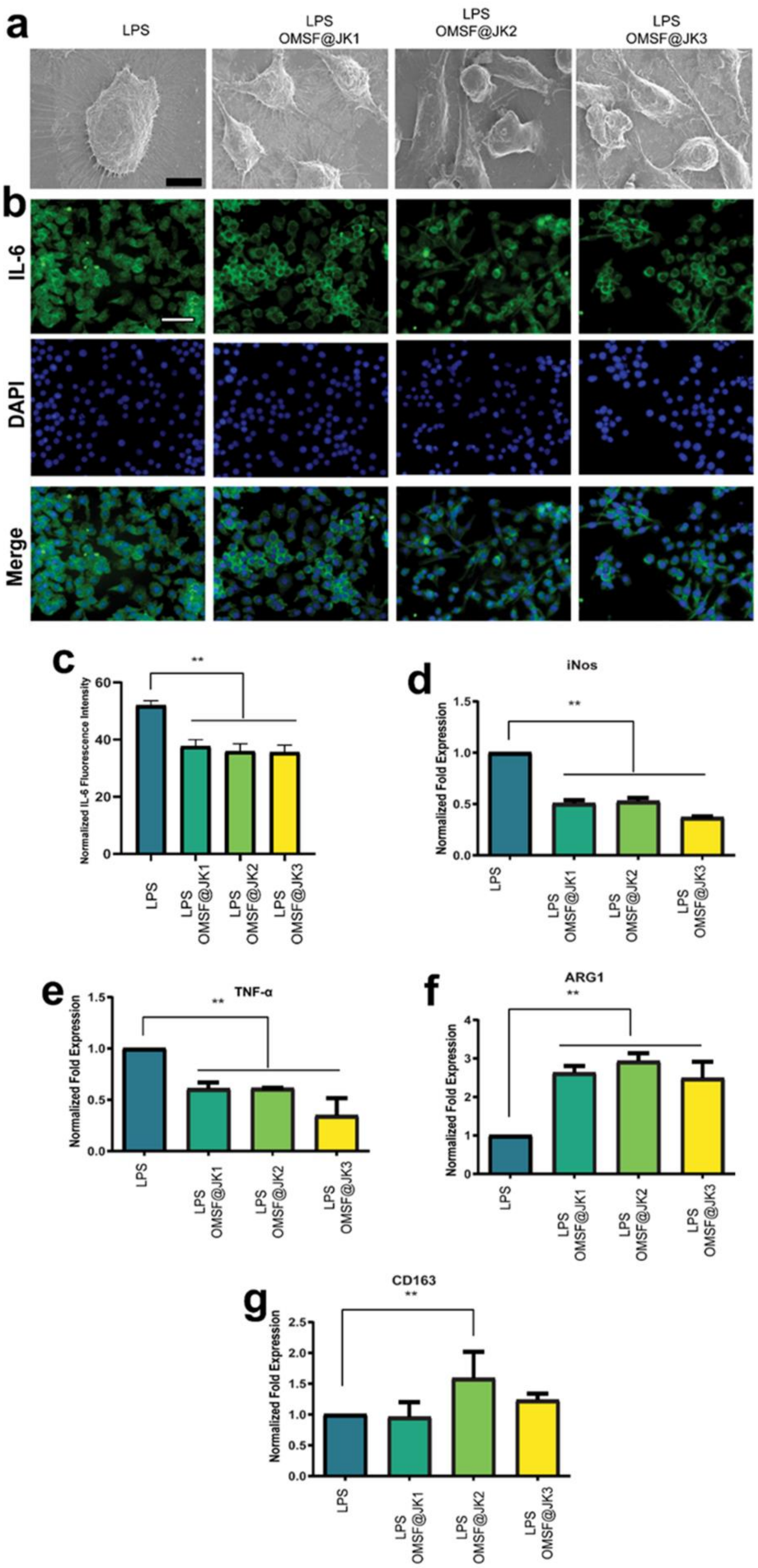

Fig. S7: Anti-inflammatory properties of nanoparticles on Macrophages: (A) Raw co-cultured with nanoparticles for 24 h with LPS activated were prepared and examined by SEM (scale bar: 20 μm). (B) Expression of IL-6 (green) (Scale bar: 100 μm), a proinflammation marker, was chosen to stain the

Raw cultured in nanoparticles for 24 h with LPS activated. The positive expression of IL-6 presented inflammation of the RAW. Cellular nuclei were visualized with 4′, 6- diamidino-2-phenylindole (DAPI) (blue). (C) The fluorescence intensity of IL-6 was quantified and compared with the LPS group. The IL-6 expression was significantly lowest in treatment groups. (D) PCR gene expression of iNOS and TNF-α as M1-like, and ARG1 and CD163 as M2-like macrophage markers were chosen to stain the LPS activated macrophages cultured with nanoparticles for 24 h. Gene expression iNOS and TNF-α was quantified and compared with LPS. The expression genes were significantly lowest in treatment groups. By contrast, the M2-like macrophage marker genes ARG1 and CD163 were unregulated in the OMSF@JK -treated groups at time points. *p < 0.05, **p < 0.01

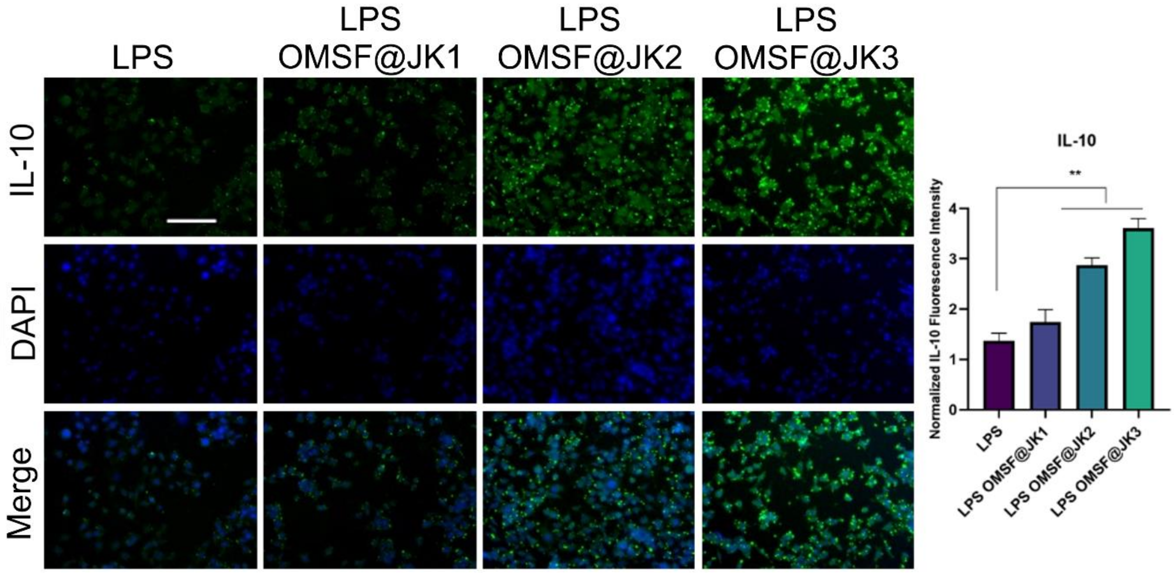

Fig. S8: Anti-inflammatory properties of JK loaded OMSF nanoparticles on Macrophages were assessed through quantification of expression of IL-10 by confocal microscopy (green). Cellular nuclei were visualized with 4′, 6- diamidino-2-phenylindole (DAPI) (blue). (C) The fluorescence intensity of IL-10 was quantified and compared with the LPS group. The IL-6 expression was significantly higher in the treatment groups and varied according to JK dose indicating that the particles are able to reduce the inflammation. **p < 0.01

S2.7: In-vitro H_2S release

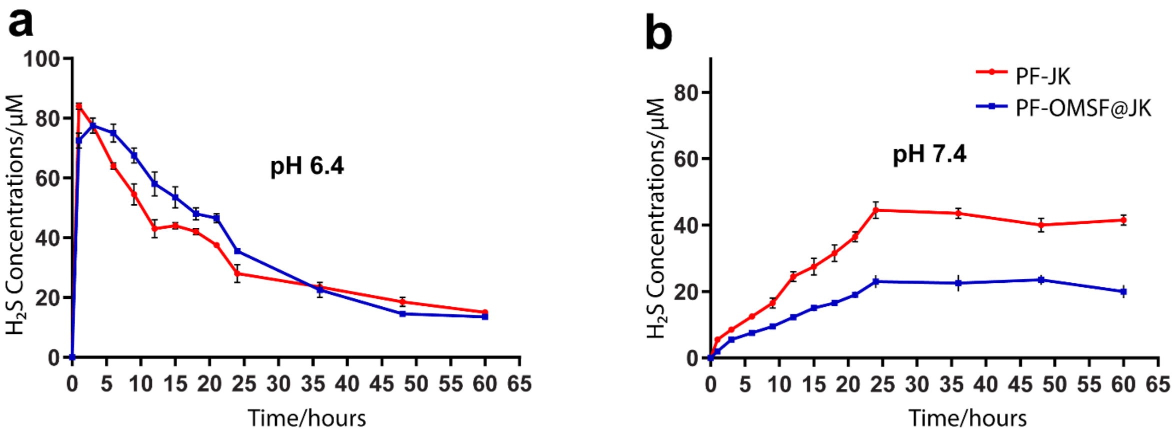

Fig. S9: The release profile of the hydrogels: Effects of pH on H_2S release from PF-JK and PF-OMSF@JK at (a) pH 6.4 and (b) pH 7.4.

S2.8: Biocompatibility of hydrogels

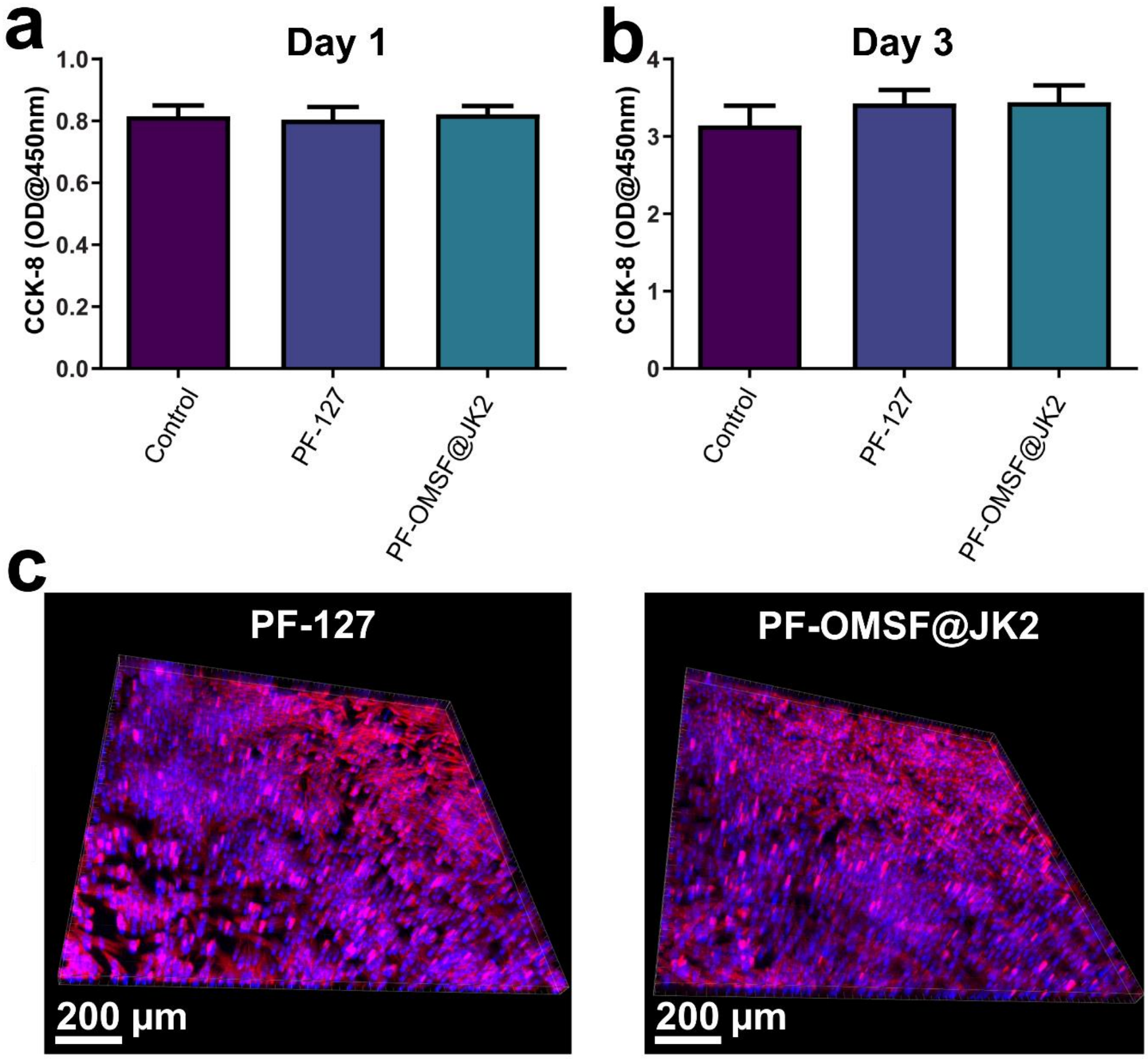

Fig. S10: Biocompatibility properties of hydrogels: The cell viability of DPSCs treated with PF127 and PF-OMSF@JK as well as the untreated control was measured by CCK-8 assay at (a) day 1 and (b) day 3. Data are shown as mean ± SD. *p < 0.05, **p < 0.01. B. A 3D LSCM visualization of DPSCs after one day of culture inside the hydrogels, DPSCs were labeled by 1 mg/mL of phalloidin-TRITC and 2 mg/mL DAPI. DPSCs in PF-OMSF@JK2 hydrogel proliferation in similar to the PF-127, the porous size of DPSCs in the range 50–150 μm was visualized by using confocal laser scanning microscopy.

S2.9: Histology of the spinal cord sections

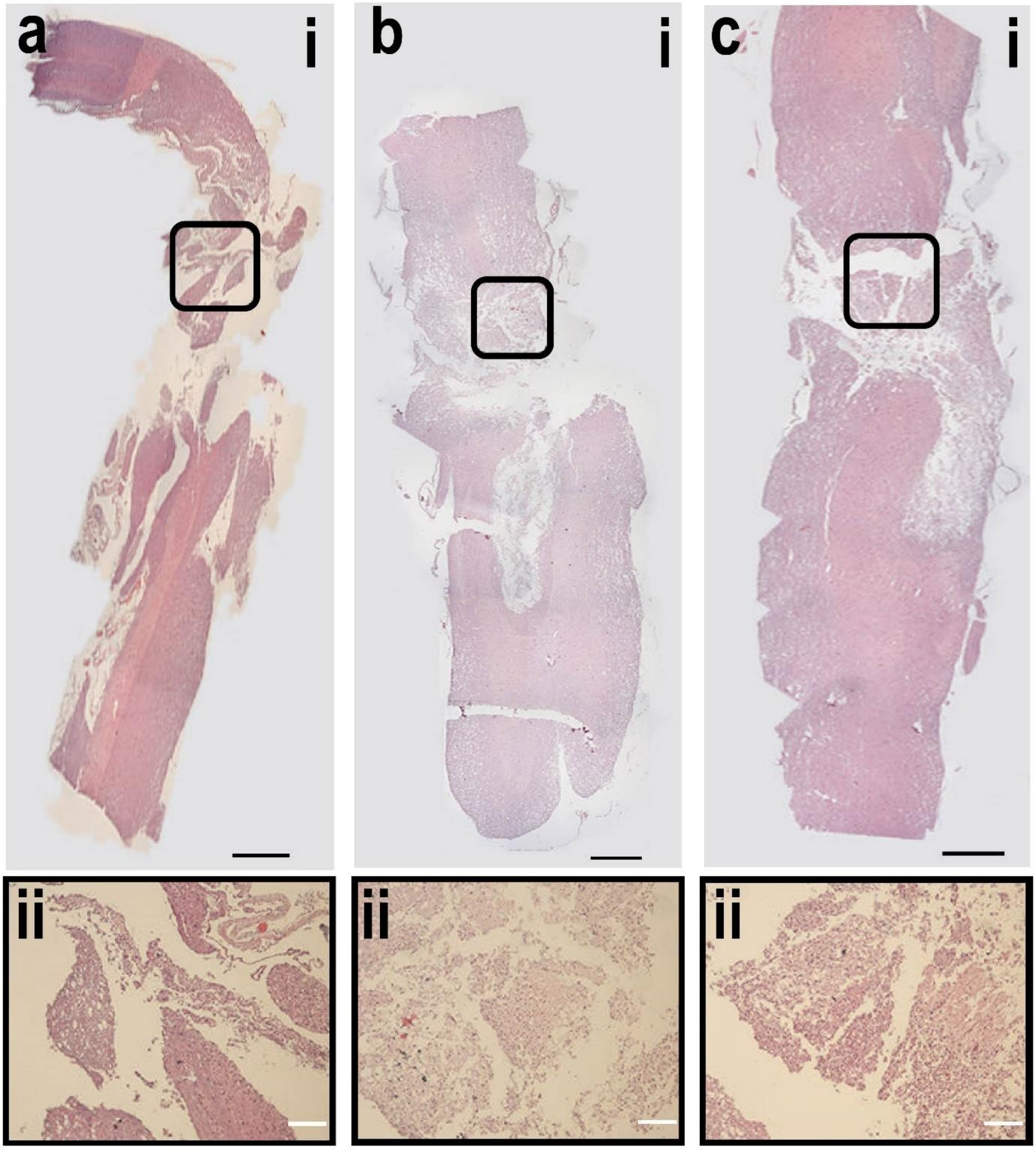

Fig. S11: Hematoxylin and eosin (H&E) stained whole spinal cord sections (top panels, i) and corresponding high-magnification images (bottom panels, ii). (a i) Representative H&E stained whole spinal cord section of SCI control (i.e. no treatment) group while a high-resolution image corresponding to the epi-center of the SCI is shown in (a ii). (b i) H&E stained whole spinal cord section of SCI rat treated with PF-OMSF/DPSCs group with a high-resolution image corresponding to the epi-center of the SCI is shown in (b ii). (c i) H&E stained whole spinal cord section of SCI rat treated with PF-OMSF@JK2/DPSCs group with a high-resolution image corresponding to the epi-

center of the SCI is shown in (c ii). The scale bar for the top panel (i.e. whole spinal cord section) is 2.5 mm, whereas the scale bar for high-resolution images in the bottom panel is 200 µm. In the top panel, the proximal end of the spinal cord is at the top, while the distal end is at the bottom of the image.
